# Effect of developmental dynamics on WRKY expression in barley with varying phenologies and trichome micromorphologies

**DOI:** 10.1186/s12870-025-07933-5

**Published:** 2025-12-17

**Authors:** Piotr Ogrodowicz, Anetta Kuczyńska, Krzysztof Mikołajczak, Michał Kempa, Dominika Maryniak, Martyna Michałek, Paweł Krajewski, Jolanta Belter, Magdalena Gawlak, Hazem M. Kalaji, Piotr Dąbrowski, Jacek Mojski, Iwona Belusiak

**Affiliations:** 1 Institute of Plant Genetics Polish Academy of Sciences, Poznań, Poland; 2 Institute of Plant Protection – National Research Institute, Poznań, Poland; 3 Institute of Technology and Life Sciences - National Research Institute, Raszyn, Poland; 4 Department of Plant Physiology, Institute of Biology, Warsaw University of Life Sciences—SGGW, Warsaw, Poland; 5 Institute of Environmental Engineering, Warsaw University of Life Sciences—SGGW, Warsaw, Poland; 6 The Foundation of Green Infrastructure, Lukow, Poland; 7 RIVBIO sp. z o. o, Wrocław, Poland

**Keywords:** Barley, Drought resistance, Earliness, *Fusarium* resistance, Multiple stress combinations, Trichome morphology

## Abstract

**Background:**

Understanding how developmental traits and epidermal structures contribute to stress tolerance is essential for improving crop resilience. In this study, four spring barley genotypes differing in phenology and epidermal features—CamBW1, CamWa1, LubBW1, and LubWa1—were evaluated under combined abiotic (mild and severe drought) and biotic (*Fusarium* infection) stress, with growth regulators application.

**Results:**

The first phase of the study focused on assessing the phenological diversity of the examined barley genotypes in order to characterize the role of earliness in response to combined abiotic and biotic stresses. Phenological analysis confirmed distinct developmental patterns between early- and late-heading genotypes, supporting the presence of a “drought escape” strategy in early-flowering lines. The expression of the key photoperiod gene *HvPRR37* further reflected these phenological differences. Through a comprehensive screening of drought responses and *Fusarium* infection severity, we were able to determine the performance and stress tolerance of the studied genotypes, and subsequently explore the expression patterns of key transcription factors (WRKY). Scanning electron microscopy revealed clear variability in trichome micromorphology between genotypes: LubBW1 developed numerous small trichomes, whereas LubWa1 produced fewer but larger structures. These differences may underlie distinct responses to *Fusarium* infection, as LubBW1 trichomes appeared to trap fungal conidia more effectively. Photosynthetic performance analyses indicated that combined stress strongly affected photosystem II and I efficiency, with the most severe inhibition observed under drought + *Fusarium* + gibberellin application treatment. However, mild drought induced only moderate changes, suggesting adaptive physiological regulation. Gene expression profiling of WRKY34, WRKY51, and WRKY70 demonstrated genotype-specific and stress-intensity-dependent regulation. WRKY51 and WRKY70 responded rapidly to drought and pathogen stress, while WRKY34 expression appeared developmentally regulated.

**Conclusion:**

These results highlight the complex interplay between phenology, trichome micromorphology, photosynthetic efficiency, and transcriptional regulation in shaping barley responses to combined stresses, underscoring the developmental stage as a critical determinant of resistance activation.

**Supplementary Information:**

The online version contains supplementary material available at 10.1186/s12870-025-07933-5.

## Background

As sessile organisms, plants face a wide range of environmental stressors, including drought, extreme temperatures, nutrient deficiencies, and biotic factors such as pathogen attack and herbivory [1]. These stressors may occur individually or in combination, and plants have evolved sophisticated mechanisms to perceive and respond to such challenges. As noted by Kamran et al. [2], this involves extensive crosstalk between abiotic and biotic stress signaling pathways, with chloroplast retrograde signaling playing a central role in coordinating adaptive and cross-tolerance responses. Global warming has led to the simultaneous occurrence of various abiotic and biotic stresses in plants, prompting distinct stress responses. The multifactorial stress combination (MFSC) refers to the simultaneous occurrence of multiple environmental stressors, such as drought, heat, salinity, and pathogen attacks. Combined stress conditions elicit intricate physiological and molecular responses in plants, distinct from those caused by single stress factors. These combined stresses activate unique signaling and transcriptional networks, reflecting non-additive interactions that enable plants to acclimate to multifactorial environments [3]. Drought is one of the most significant abiotic stress factors limiting plant growth and development worldwide [4]. Water scarcity, whether caused by natural climatic conditions or human activity, can severely impair plant functions, reduce agricultural productivity, and impact ecosystems [5].

Plants activate specific and unique stress responses when exposed to multiple stress factors, and understanding these multifactorial interactions is crucial because they profoundly affect plant health, growth, and survival. When plants face a combination of biotic and abiotic stresses, the overall impact can be (i) synergistic—the combined stresses amplify each other’s negative effects (e.g., drought combined with heat stress) [6]; (ii) antagonistic—one stress reduces the effect of the other (e.g., drought stress triggering defense mechanisms that help fight off pathogens) [7]; and (iii) additive—the effects of the stresses are independent, and their combined impact equals the sum of their individual effects [8]. A critical aspect of the plant stress response is the ability to differentiate between short-term and long-term stress effects. Short-term stress can be partially mitigated by acclimation, adaptation, and repair mechanisms, whereas prolonged or severe stress conditions may lead to significant damage or even plant death [9].

Fusarium head blight (FHB) is an economically devastating disease of small-grain cereal crops caused by a complex of toxigenic *Fusarium* spp [10]. One of the major challenges in the study of resilience to multiple stresses is the rapid change in plant development. In many plant–pathogen interactions, resistance expression depends on the plant’s developmental stage at infection. Resistance may develop gradually during the life cycle and is often linked to major transitions, such as the juvenile/adult transition during vegetative growth or the flowering stage [11]. The superficial tissues (epidermis) and structures (cuticles and trichomes) of plant organs play crucial protective roles against multiple biotic and abiotic stresses. Some studies suggest that trichomes can support fungal infections by allowing spores/hyphae to attach to their surfaces, promoting further colonization [12]. However, many studies have shown that trichomes serve as a physical barrier against insects, herbivores, fungal infections, and parasitic plants [13]. The role of trichomes in fungal infections remains controversial, as the view that they facilitate fungal entry may be biased [14]. Nonetheless, their role in fungal spread could be critical, especially under additional stress. The epidermis and structures such as the cuticle and trichomes act as the primary barriers in plant–environment interactions, playing pivotal protective roles against diverse abiotic and biotic stresses. Recent studies have shown that trichomes can inadvertently support fungal infections by providing attachment points for spores/hyphae, enabling further plant colonization [14]. Despite the essential role of the aerial epidermis in stress interactions, the precise influence of its structural arrangement on plant defense mechanisms, particularly during development, remains incompletely understood.

Recent integrative studies combining precision phenotyping, morpho-physiological assessments, and transcriptomic profiling have substantially advanced our understanding of barley adaptation to drought stress. These approaches have revealed numerous novel drought-responsive genes and provided new insights into the molecular and physiological mechanisms underlying stress resilience. Transcriptomic analyses under drought and subsequent rewatering identified genes involved in chlorophyll maintenance, photosynthetic efficiency, and gibberellin biosynthesis linked to stress adaptation [15, 16]. Collectively, these findings highlight the complex, multilayered regulation of drought tolerance in barley and provide valuable genomic resources for breeding more resilient cultivars.

Chlorophyll *a* fluorescence is widely used to assess the photosynthetic performance of plants, especially those under stress [17]. This method can provide insights into the efficiency of photosystem II (PSII), which is critical for the light-dependent reactions of photosynthesis [18]. During photosynthesis, the light energy absorbed by chlorophyll is utilized in three ways: photochemistry (photosynthesis), heat dissipation, and chlorophyll fluorescence [19]. The induction of chlorophyll fluorescence is a widely used method in photosynthesis research, as it is noninvasive, very sensitive, quick, easy to measure, and contains important information about the photosynthetic apparatus. The fluorescence varies between the initial level (F_0_) and the maximum level (F_M_) of the curve, which is called O-J-I-P [20]. Some of the parameters calculated via the O-J-I-P test are related to energy fluxes for light absorption (ABS), trapping (TR) of the excitation energy, and electron transport (ETR) per reaction center (RC) or per sample area called the cross section (CS).

A key focus of this research is the analysis of WRKY transcription factor expression, which plays a vital role in regulating plant responses to both biotic and abiotic stresses [21, 22]. In barley (*Hordeum vulgare* L.), WRKY transcription factors are key regulators of stress-induced gene expression, helping the plant adapt to challenging environmental conditions. WRKY proteins regulate the expression of pathogenesis-related (PR) proteins involved in defense, including antimicrobial activity and cell wall strengthening [23]. Barley WRKYs increase resistance to fungal pathogens such as *Blumeria graminis* (causing powdery mildew) [24] and *Fusarium* spp [25]. In our study, we selected three transcription factors (TFs) representing two distinct subgroups, to illustrate differences in their expression patterns under various stress conditions.

This study utilized lines derived from crosses between Bowman eceriferum (*cer*) mutants with glaucous or nonglaucous phenotypes and early-heading or late-heading barley varieties. Eceriferum genes are known primarily for their role in cuticular wax biosynthesis and deposition, which can indirectly affect trichome development [26]. Barley trichomes are typically covered with cuticular wax, enhancing their protective functions. Mutations in Eceriferum genes may reduce wax production, leading to trichomes with diminished effectiveness as barriers. Although Eceriferum genes are associated mainly with wax, some mutations could influence trichome development, density, or structure, as wax biosynthesis and trichome formation involve overlapping genetic pathways [27].

The objective of this study was to investigate how phenological diversity and epidermal traits, particularly trichome micromorphology and wax deposition, influence barley responses to combined abiotic (drought) and biotic (Fusarium head blight) stress. To achieve this, we used four contrasting DH barley lines differing in heading time (early- and late-heading) and wax coverage to assess their susceptibility to drought and *Fusarium* infection. Ultimately, the main goal of the study was to analyze the dynamics of WRKY transcription factor expression in plants exposed to multifactorial stress conditions. We hypothesize that the structural and phenological diversity among these lines, which differ in stress resistance, contributes to the differential activation of WRKY transcription factors, thereby shaping plant resilience under combined abiotic and biotic stresses.

## Materials and methods

### Plant materials

Four spring barley lines derived from crosses between Bowman eceriferum (*cer*) mutants with glaucous or nonglaucous phenotypes and early/late heading genotypes (a Syrian breeding line and an old Polish cultivar) [28, 29] were examined in this study. Lubuski is an old Polish cultivar derived from a Heines–Haisa/Skrzeszowicki hybrid. Cam/B1/CI08887//CI05761 (referred to as CamB) is a Syrian breeding line provided to Dr. A. Górny (IPG PAS) by Drs. S. Grando and S. Ceccarelli of ICARDA in Aleppo. Wa1 is a reference variety known for its strong wax (obtained from the Research Centre for Cultivar Testing collection, Poland). BW408 (referred to as BW1) is a Bowman backcross-derived line (NGB 110915), Eceriferum-s.31, glossy, with close linkage between alleles at the *gsh5* locus and *Eam6* (Early maturity 6) (from the Nordic Genetic Resource Center collection, Sweden). The experiment used the following 4 DH lines: CamBW1: early-heading, glossy line derived from a cross between CamB × BW408; CamWa1: early-heading, glaucous line derived from a cross between CamB × Wa1; LubBW1: late-heading, glossy line derived from a cross between Lubuski × BW408; and LubWa1: late-heading, glaucous line derived from a cross between Lubuski × Wa1.

### Experimental setup

**Fig. 1:**
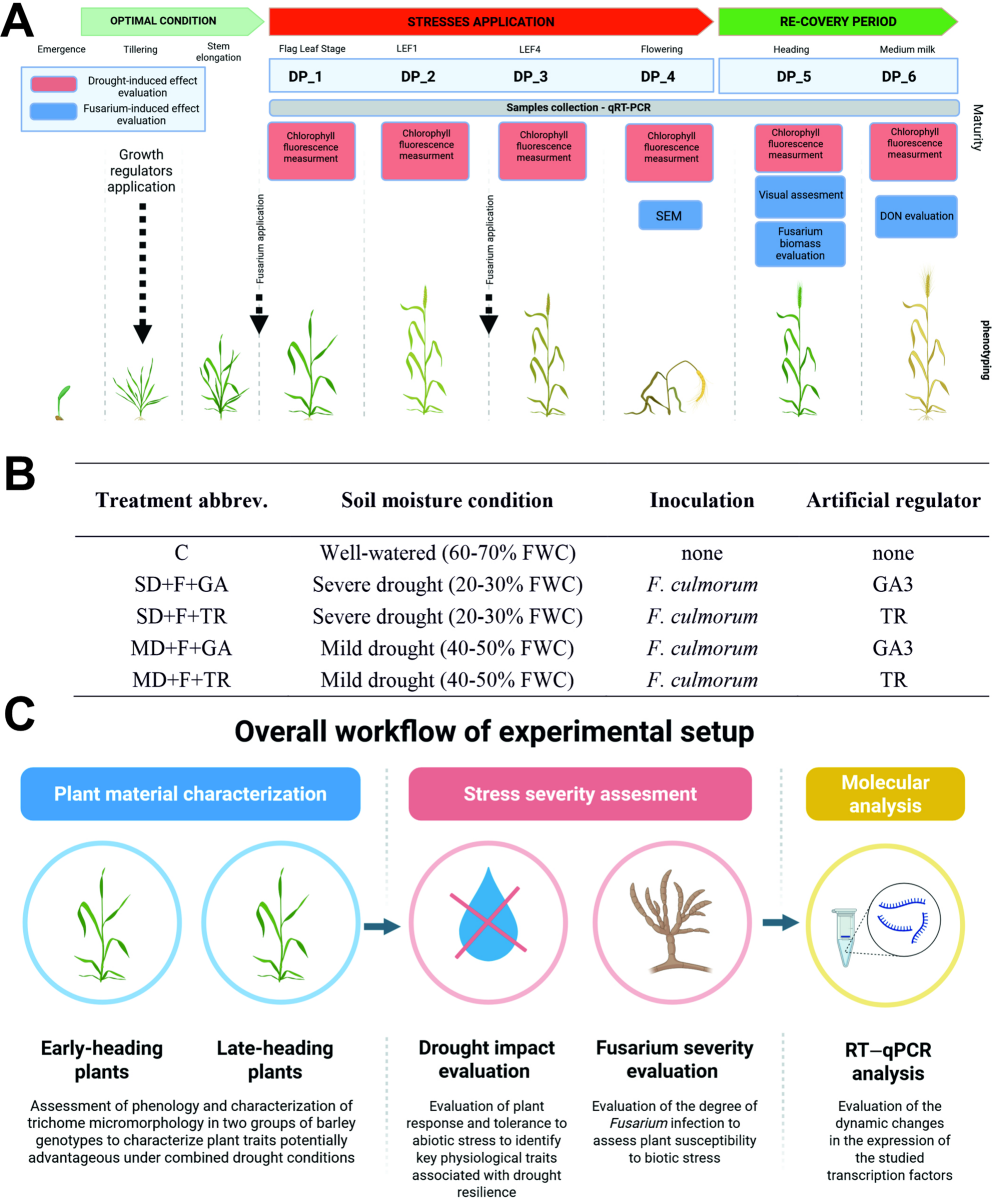
Experimental setup and stress-induced changes evaluation in barley genotypes. **A** Experimental setup with a description of the time of stress-induced change evaluation. The red and blue squares indicate the results of the drought-induced effect evaluation assays and the *Fusarium*-induced effect evaluation assays, respectively. Samples were collected at four developmental stages during the stress period: the flag leaf stage (Z37, on the Zadoks scale) (Development point 1—DP_1), LFE1 (DP_2), LFE4 (DP_3), and flowering (Z65) (DP_4). For the rewatering period, two additional developmental points were defined: DP_5 (heading) (Z55) and DP_6 (medium milk) (Z75). **B** Treatment description (with abbreviations). **C** Roadmap of the experimental design. The figure was created using BioRender (www.biorender.com)

The experiment was arranged in a completely randomized design with three pots per treatment and genotype, each pot containing five plants representing five biological replicates. All the experiments were conducted in growth chambers under fully controlled conditions (IPG PAS phytotrons). The study followed the Barley External Developmental Stages scale [30], where the later stages of the Zadoks scale were replaced by the last flag extension (LFE) stages, offering a clear system for observing developmental stages related to reproductive development. The samples were collected at six developmental stages (Fig. 1).

### Soil moisture regime

The plants were irrigated until the flag leaf stage (Z37). The plants were then subjected to three irrigation treatments: (i) well-watered treatment (soil maintained at ~ 70% of field water capacity (FWC)); (ii) mild drought stress (10 days) (at 40–50% FWC); and (iii) severe drought stress (10 days) (at 20–30% FWC). The soil moisture in each pot was controlled gravimetrically by weighing and volumetrically, following the method described by Ogrodowicz et al. [28], to maintain the targeted control and drought conditions.

### Application of artificial growth regulators

GA3 (gibberellic acid) (SERVA, Electrophoresis GmbH, Heidelberg, Germany) solution was sprayed directly onto the leaves at the tillering stage (Z21). Trinexapac-ethyl (TR) was applied as a commercial product, Moddus 250 EC (Syngenta, Greensboro, NC, USA), at the tillering stage. Detailed descriptions of the artificial growth regulator preparation are provided by Ogrodowicz et al. [29].

### Inoculum preparations and applications

*Fusarium culmorum* isolates were incubated on wheat grain (50 g) in 300 mL Erlenmeyer flasks for five weeks, as described by Ogrodowicz et al. [31]. Briefly, colonies were covered with 15 mL of sterile distilled water. The inoculum was prepared just before inoculation via liquid cultures of *F. culmorum* (isolate KF846) and 0.0125% TWEEN (Sigma–Aldrich Chemie GmbH, Steinheim, Germany). The inoculum concentration was adjusted to 105 spores/ml. Treatment descriptions and abbreviations are presented in Fig. 1.

### Phenology and phenotypic evaluation

Phenological observations (developmental stages: tillering, flag leaf stage, flowering, and heading) and 12 yield-related traits were analyzed in this study. Phenological observations were conducted based on monitoring all pots within each treatment, whereas the exact number of replicates used for biometric measurements is provided in Table S1.

### Evaluation of the effects of drought

Chlorophyll *a* fluorescence measurements.

The M-PEA-2 chlorophyll fluorescence measurement system (Multifunction Plant Efficiency Analyzer, Hansatech, UK) was used in this study, following the protocol described by Dąbrowski et al. [32]. The following chlorophyll fluorescence signals were measured: prompt chlorophyll *a* fluorescence (PF), delayed chlorophyll *a* fluorescence (DF), and 820-nm light reflection (MR_820_). Measurements were performed using nine replicates.

The JIP test described by Strasser et al. [33] was used to recalculate the characteristic points of photoinduced chlorophyll fluorescence curves into specific parameters of the light phase of photosynthesis. Descriptions of the fluorescence parameters are provided in Table 1. To better visualize the influence of multiple stresses on chlorophyll fluorescence dynamics, the relative variable fluorescence was calculated. In the next stage, differences in the relative variable fluorescence curves were calculated by subtracting the normalized fluorescence values (between the O and P steps) recorded in the control and stressed plants.

As recommended by Goltsev et al. [34], the characteristic points (I_1_, I_2_, and D_2_) of the delayed fluorescence (DF) curve were also assessed. The I_1_ point is the first maximum of the curve, I_2_ is the second maximum, and D_2_ is the second minimum. Two ratios were calculated: (I_1_ − D_2_)/D_2_ and I_1_/I_2_. The modulated reflection (MR) signal was measured simultaneously with the DF. MR_0_ represents the 820-nm reflection at the onset of actinic illumination. The minimum (MR_min_) and maximum (MR_max_) levels were also calculated, as were ∆MR_fast_ (the difference between MR_0_ and MR_min_) and ∆MR_slow_ (the difference between MR_max_ and MR_min_).

**Table 1:** The description of fluorescence parameters

Parameter	Description
ABS/RC	Absorption flux (exciting PSII antenna Chl *a* molecules) per RC, ABS/RC = M_0_ × (1/V_j_) × (1/ϕ_Po_)
TR_0_/RC	Trapped energy flux (leading to Q_A_ reduction), per RC, TR_0_/RC = M_0_ × (1/V_j_)
ET_0_/RC	Electron transport flux (further than Q_A_^−^), per RC, ET_0_/RC = M_0_ × (1/Vj) × (1 − Vj)
RE_0_/RC	Electron flux reducing end electron acceptors at the PS1 acceptor side, per RC, RE_0_/RC = M_0_ × (1/V_j_) × (1 − V_i_)
DI_0_/RC	Dissipated energy flux per RC, DI_0_/RC = ABS/RC – TR_0_/RC
ϕ_Po_	Maximum quantum yield for primary photochemistry, ϕ_Po_ = 1 − (F_0_/F_M_)
ψ_Eo_	Efficiency/probability that an electron moves further than Q_A_^−^, ψ_Eo_ = 1 − V_j_
ϕ_Eo_	Quantum yield for electron transport, ϕ_Eo_ = [1 − (F_0_/F_M_)] × (1 − V_j_)
δ_Ro_	Efficiency/probability with which an electron from the intersystem electron carriers is transferred to reduce end electron acceptors at the PSI acceptor side, δ_Ro_ = (1 − V_i_)/(1 − V_j_)
ϕ_Ro_	Quantum yield for reduction of end electron acceptors at the PSI acceptor side, ϕ_Ro_ = [1 − (F_0_/F_M_)] × (1 − V_i_)
ϕ_Do_	Quantum yield of energy dissipation, ϕ_Do_ = 1 -ϕ_Po_
RC/ABS	RCs per PSII antenna Chl *a* molecule (reciprocal of ABS/RC)
γ_RC_	Probability that a PSII Chl *a* molecule functions as RC, γ_RC_ = RC/(ABS + RC) = RC/ABS/(1 + RC/ABS)
RC/CSo	Density of RCs (Q_A_ reducing PSII reaction centers) = Po (V_J_/M_0_) F_0_
PI_ABS_	Performance index for energy conservation from photons absorbed by PSII until the reduction of intersystem electron acceptors, PI_ABS_ = [γ_RC_/(1 - γ_RC_)] × [ϕ_Po_/(1 - ϕ_Po_)] × [ψ_Eo_/(1 - ψ_Eo_)]
PI_total_	Performance index for energy conservation from photons absorbed by PSII until the reduction of PSI end electron acceptors, PI_total_ = PI_ABS_ × [δ_Ro_/(1 - δ_Ro_)]

### Chlorophyll, flavonol, anthocyanin, and nitrogen balance indices

The leaf chlorophyll index (Chl), flavonol index (Flav), anthocyanin index (Anth), and nitrogen balance index (NBI) were determined via Dualex Scientific+ (Force-A, Orsay, Paris, France). The chlorophyll content was calculated from the far-red light absorbed by chlorophyll and the transmittance of near-infrared light as a reference. The flavonoid and anthocyanin contents were calculated from the different ratios of chlorophyll fluorescence in the leaf epidermis. The nitrogen balance index is the ratio of chlorophyll to flavonoids. Measurements were taken at six developmental points on the most recently fully expanded leaf, as described by Cerovic et al. [35]. Measurements were performed using nine replicates.

### Evaluation of the effects of fusarium infection

#### *Fusarium *visual assessment

Visual evaluation of *F. culmorum* infection followed the protocol described by Mesterházy et al. [36] and was performed as follows:

*Fusarium* VE (%) = (number of infected spikes/number of all spikes from the plant) * 100.

#### Fungal biomass evaluation

*Fusarium* biomass was evaluated following the methodology of Perlikowski et al. [37], with modifications. Briefly, mycelia of the *F. culmorum* isolate KF 846 were grown on potato dextrose agar (PDA) supplemented with streptomycin for 5 days at room temperature. DNA was extracted via the CTAB method: lyophilized fungal tissue was frozen, ground, incubated with CTAB, and subjected to chloroform: isoamyl alcohol extraction. DNA was precipitated with isopropanol and sodium citrate, washed with ethanol, dried, and dissolved in TE buffer. Real-time PCR was performed via a CFX96 touch device (Bio-Rad) with specific primers and probes for the ACT2, TUBG, and Fc01 genes in a 20 µL reaction volume. The PCR profile included initial denaturation (95 °C, 5 min) followed by 45 cycles of denaturation (95 °C, 10 s) and annealing/extension (60–62 °C, 25 s), and fluorescence was detected in the FAM channel.

#### Estimation of deoxynivalenol (DON) content

The DON content was evaluated according to the methodology presented in Ogrodowicz et al. [31]. Briefly, the DON content (ppm) in infected grain samples (three replicates) was measured via the Ridascreen^®^ DON competitive ELISA kit (R-Biopharm AG, Darmstadt, Germany) following the manufacturer’s protocol. Five grams of ground kernels were mixed with 100 ml of distilled water and shaken manually for 3 min. After filtration through Whatman No. 1 paper, 50 µl of filtrate was added to each well. The absorbance was read at 450 nm with a microplate reader, and the data were analyzed via RIDA^®^SOFT Win software.

### Scanning electron microscopy (SEM)

Chaffs were air-dried under ambient conditions immediately after collection. Sample fragments were then attached to stubs via double-sided adhesive carbon discs. The samples were coated with gold/palladium and photographed *via* a scanning electron microscope (Hitachi S-3000 N SEM) equipped with a secondary electron detector (SE). The trichome densities of the studied genotypes were determined as the number of trichomes per unit area (mm^2^). All the samples were tested in five replications.

### Image analysis of trichome morphology

Trichome micromorphology was determined via the Image Analyzing System Motic Images Plus 3.0, partially following the protocol described by Mirnezami et al. [38], with modifications. Briefly, the invert command was used to invert the image colors (Fig. S1). Measurements were then taken *via* the quantitative anatomical data image command Auto Calculation, which provides detailed data about the segmented objects from the image, including the number of objects, percentage of object area in the picture area, maximum object area, mean object area, maximum object perimeter, and mean object perimeter (Table S2).

### RT‒qPCR analysis

Chaffs were collected at six developmental stages (Fig. 1). Four biological replicates were collected for each DP. The plant tissues were immediately frozen in liquid nitrogen and stored at − 80 °C until RNA extraction was performed. The RNA was extracted via a RNeasy Mini Kit (QIAGEN, Hilden, Germany) according to the manufacturer’s protocol with on-column DNase treatment (QIAGEN, Hilden, Germany). Additionally, all the isolated RNA samples were treated with TURBO DNase (Thermo Fisher Scientific, Vilnius, Lithuania) according to the manufacturer’s instructions to exclude trace contamination of the samples with genomic DNA. The purities of all the RNA samples were assessed *via* OD260/280 and OD260/230 absorbance ratios, whereas their structural integrity was evaluated by denaturing agarose gel electrophoresis.

In addition to the selected WRKY genes, the expression pattern of HvPRR37 (*Ppd-H1*) was also analyzed to characterize the phenological differences among the studied plant materials. The expression of the investigated genes (WRKY34, WRKY51, WRKY70, HvPRR37) was normalized *via* two stable reference genes (UBI—GenBank ID: M60175.1; ACT1—GenBank ID: AY145451.1) [39], and the stability of the reference genes in the experimental setup was confirmed *via* a tool (Reference Gene Selection Tool - Bio-Rad CFX Maestro Software), that supports the geNorm algorithm (Table S3). The methodology for analyzing the expression of selected genes has been described in detail in Ogrodowicz et al. [29]. Briefly, gene expression was analyzed via RT‒qPCR via the CFX Connect Real-Time PCR Detection System and iTaq Universal SYBR Green One-Step Kit (Bio-Rad Laboratories, Hercules, CA, USA) following the manufacturer’s protocol. Each 10 µL reaction contained 500 nM primers and 1 µg of RNA. The expression data were averaged from three technical replicates across four biological replicates. Amplification specificity was verified by melting curve analysis (63–95 °C, 0.5 °C increments), and RT‒qPCR products were sequenced (AMU, Poznań, Poland). Data analysis was performed *via* CFX Maestro v2.0, and the RT‒qPCR analysis met the MIQE criteria [40].

### Statistical analysis

Analysis of variance (ANOVA) for observed quantitative traits was performed in a model containing the fixed effects of genotype (CamBW1, CamWa1, LubBW1, LubWa1 - G), treatment (C, SD + F + GA, SD + F + TR, MD + F + GA, and MD + F + TR - T), and the G × T interaction. For dynamic traits (observed at all six developmental stages), ANOVA was performed for DP_4 and DP_6 for each genotype separately for the factors treatment, DP and their interaction.

Significant sources of variation in ANOVA were selected at *p* < 0.001 (approximate threshold resulting from the application of the Bonferroni correction concerning multiple testing for all traits). Multiple comparisons of mean values were made *via* Fisher’s protected least significant difference (FPLSD) test at *p* < 0.05. Statistical computations and visualizations were performed in Genstat 22 [41].

## Results

### Plant phenology and trichome micromorphology

#### Phenology

**Fig. 2:**
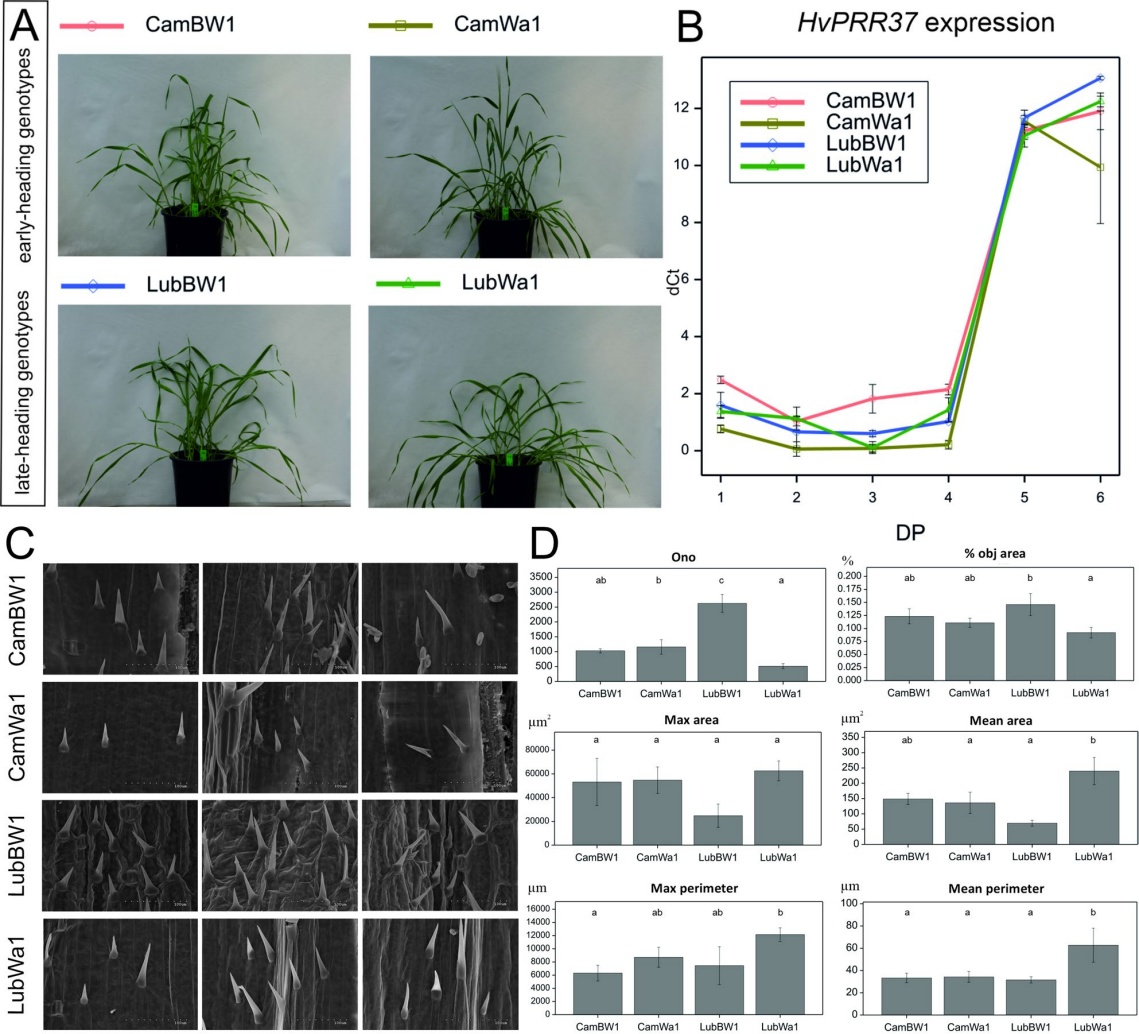
Phenological differentiation and trichome morphology in barley genotypes. **A** Phenological differentiation of the studied barley genotypes; on the basis of observations of the length of the vegetative period, two groups of plants could be distinguished: (i) early-heading and (ii) late-heading genotypes. The presented photographs, taken at the same time, show plants that have reached different developmental stages: early-heading plants are in the flag leaf stage (Z38), and late-heading plants are still at the end of the tillering stage (Z29). **B** Expression profiles of the key photoperiod gene (*HvPRR37*) in the studied forms. DP – development point. **C** Scanning electron micrographs of the trichomes of the four studied genotypes (with three repetitions). The extraordinary organization of trichome structures was observed in this study for LubBW1. **D** The variability of the mean values (with standard errors) of the trichome morphology traits of the studied plants grown under control conditions; letters indicate statistically similar mean values at *p* < 0.05 according to the Fisher least significant difference test

Analysis of variance (ANOVA) revealed significant differences (*p* < 0.001) between the studied genotypes in terms of the time taken to reach three phenological stages: the flag leaf stage (Z38), the flowering stage (Z65), and the heading stage (Z55) (Table S4). Generally, genotypes with a late-heading progenitor (Lubuski) reach the tillering stage later than those derived from crosses of the Syrian breeding line (CamBW1 and CamWa1); hence, two subgroups of the studied plants can be distinguished: early-heading and late-heading plants (Fig. 2 A). Compared with mild stress, the applied stress conditions significantly affected plant development (Fig. S2), with the number of days to flowering increasing, particularly for early-heading plants under severe drought. For late-heading plants, the days to flowering were similar under both types of drought conditions.

#### *HvPRR37* expression

The expression level of the *HvPRR37* gene was recorded for plants grown under control conditions (Fig. 2c). For all the studied genotypes, a similar pattern of gene expression was recorded, but some differences between them were observed at particular developmental stages. A relatively high level of *HvPRR37* expression was observed at the first, second, third, and fourth DPs in CamBW1 tissues. In contrast, CamWa1 presented the lowest *HvPRR37* expression at these stages. A different *HvPRR37* expression pattern was observed during DPs 5–6, with a rapid increase in expression for all genotypes after DP_4 (flowering). The highest expression level of *HvPRR37* was detected in LubBW1 at DP_6.

#### Trichome morphology

SEM image analysis revealed differences in trichome micromorphology between genotypes, with significant differences in Ono and the mean area (Fig. 2c, d, Fig. S3, Table S4). The highest values for the Ono and area traits were recorded for the LubBW1 genotype, whereas LubWa1 presented the lowest values for these traits. For trichome morphology-related traits, opposite trends were observed: LubBW1 presented the lowest values for max_area, mean_area, max_perimeter, and min_perimeter, whereas LubWa1 presented the highest values for these traits.

### *Fusarium*-induced effect evaluation

**Fig. 3:**
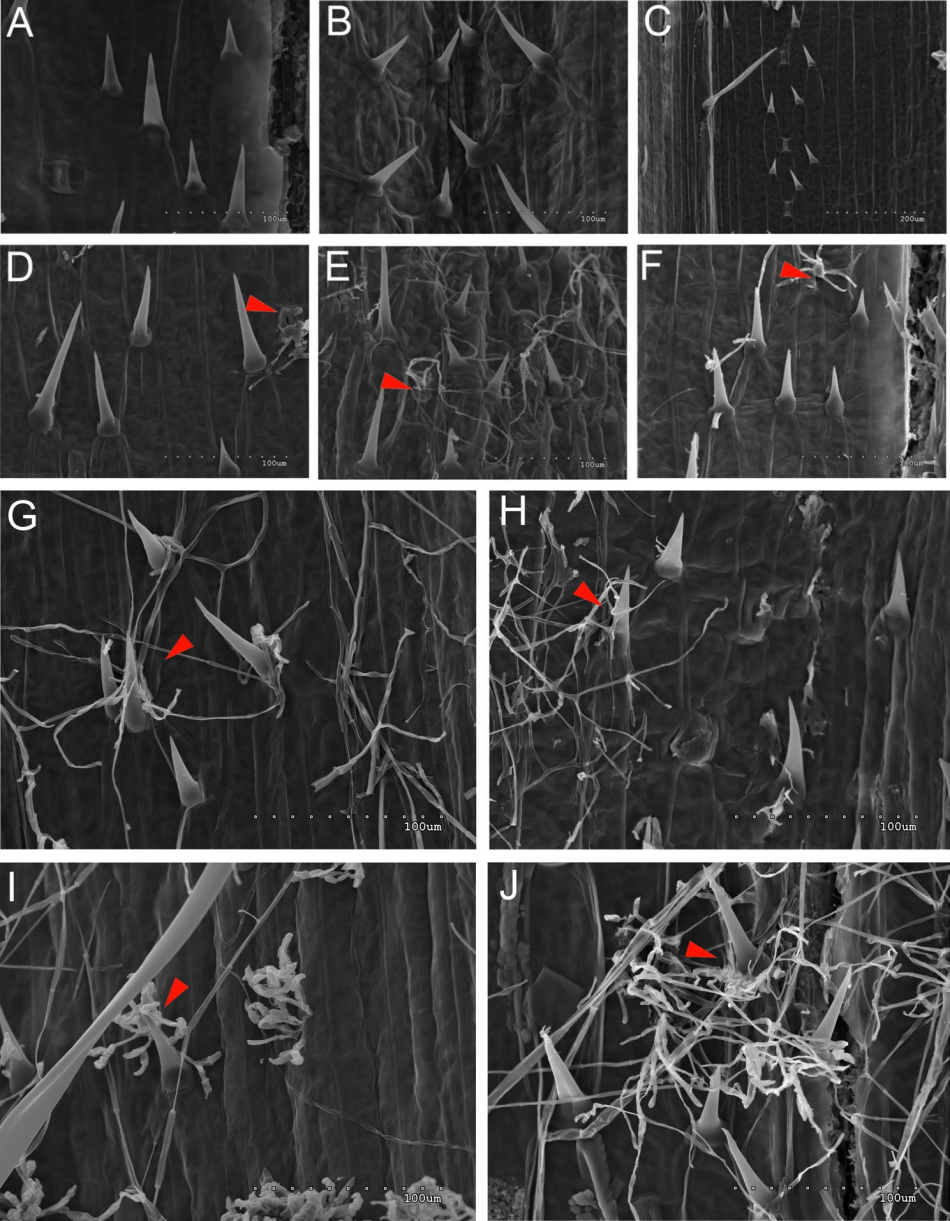
Spatial distribution of trichomes and *Fusarium* mycelium on barley stomata. Scanning electron micrographs. Spatial variations in trichome localization in the vicinity of stomata were observed for the CamBW1 (**A**), CamWa1 (**B**), and LubBW1 (**C**) genotypes grown under control conditions. Images showing the deposition of fungal mycelium (*F. culmorum*) on barley stomata upon infection observed for the following genotypes: CamBW1 (**D**), CamWa1 (**E**), and LubBW1 (**F**). Fungal hyphae coil the trichomes of CamBW1 plants subjected to SD + F + GA (**G**), MD + F + GA (**H**), MD + F + TR (**I**) and SD + F + TR (**J**) conditions. Red arrow = rapid development of *Fusarium* hyphae in the vicinity of trichomes surrounding plant stomata

ANOVA revealed no significant differences between genotypes and treatments (*p* < 0.001) for traits associated with *Fusarium* infection severity, including *Fusarium* biomass, % infected spikes, and DON content (Table S4). In some cases, no visible infestation was detected (e.g., LubBW1 grown under MD + F + TR conditions), or the biomass could not be determined (e.g., CamWa1, LubBW1, and LubWa1 under MD + F + TR conditions) (Fig. 3). *Fusarium* symptoms were consistently observed in CamBW1. In general, the highest level of infection was recorded in plants grown under MD + F + GA conditions, except for CamBW1, which presented a relatively low percentage of infected spikes. A significant effect of the G × T interaction was recorded for the trait concerning trichome density on the chaff surface (Fig. S4).

### Photosynthetic performance of plants

#### Prompt chlorophyll *a* fluorescence during the stress and recovery periods - the kinetics of chlorophyll fluorescence induction curves

The relative variable fluorescence was calculated to better visualize the effects of multiple stresses on chlorophyll fluorescence dynamics. Curves were constructed by subtracting the normalized fluorescence values (between O and K, O and J, J and P, or I and P, respectively) recorded in control plants from those recorded in plants under drought stress, as described by Dąbrowski et al. [32] (Figs. S5, S6, S7). The OJIP transients were converted into biophysical parameters in the next stage, and deviations between stressed and control plants are shown as differential curves.

DP_1 The chlorophyll fluorescence induction curves for CamBW1 grown under SD conditions presented a greater trajectory than those recorded under MD and control conditions did (Fig. S5A). In LubBW1, the curve of the SD + F + GA variant was greater than that of the control, whereas the curve of the SD + F + TR condition was lower. For LubWa1 and CamWa1, the curves of the SD + F + TR variant were lower than those of the control. DP_2 In LubBW1, the curves of MD + F + GA and MD + F + TR were lower than those of the control (Fig. S5B). In LubWa1, the MD + F + TR variant had the lowest curve distributions. In CamWa1, the curve was greater than that of the control for the MD + F + GA variant. DP_3 The SD + F + GA variant in CamBW1 presented a greater curve than the other types of treatments did, especially at points O and J (Fig. S6A). The MD + F + TR variant had the lowest curve, with differences also visible at point I. In LubWa1, the MD + F + GA variant had a higher curve than the control at points I and P, whereas the MD + F + TR variant had the lowest curve. DP_4 In all the genotypes, the MD + F + TR condition had a lower curve progression at points O, J, and I than did the control condition (Fig. S6B). For the CamBW1, LubBW1 and LubWa1 genotypes, the curves for the SD conditions had a lower progression at these points. DP_5 CamBW1 has a higher curve distribution at points O, J, and I under MD + F + GA (Fig. S7A). For LubBW1 and CamWa1, the curves had a lower course at points O, J, and I under all applied stress conditions. The curves drawn for LubWa1 plants grown under all stress conditions presented greater trends at points O and J than did those of the control plants. DP_6 At the second rewatering point, the curve distributions of the studied genotypes differed across treatments (Fig. S7B). In CamBW1, curves drawn for all applied stress conditions, except MD + F + TR, were higher than those drawn for the control condition.

#### Chlorophyll *a* fluorescence increased during the stress and recovery periods—the distribution of chlorophyll fluorescence parameters

DP_1: In CamBW1, visible changes in OJIP parameters occurred under MD + F + GA, SD + F + GA, and SD + F + TR; under these conditions, the PI_ABS_ decreased, whereas the ABS/RC and DI₀/RC increased. LubBW1 presented increased PI_ABS_ under MD + F + GA and SD + F + TR. In LubWa1, the PI_ABS_ increased under MD + F + TR. (Fig. S8). DP_2: In CamBW1, the PI_ABS_ decreased under all stress conditions. LubBW1 and LubWa1 presented increases in PI_ABS_, especially under MD + F + TR in LubWa1. CamWa1 under MD + F + GA presented increased DI₀/RC and decreased PI_ABS_. DP_3: CamBW1 presented a reduced PI_ABS_ under SD + F + GA and increased PIABS under MD + F + TR. In LubWa1, the PI_ABS_ decreased under MD + F + GA but increased under MD + F + TR. CamWa1 showed that the PI_ABS_ increased under SD + F + TR and MD + F + TR. DP_4: CamBW1 had the most pronounced changes, with increased PI_ABS_, especially under MD + F + TR. LubBW1 and LubWa1 presented moderate changes, with the PI_ABS_ increasing under severe drought but decreasing under MD + F + GA. CamWa1 showed minimal changes, except under TR-containing variants, where the PI_ABS_ increased. DP_5: PI_ABS_ increased in all the variants except MD + F + GA, which caused a decrease in CamBW1. DP_6: Mild drought led to the most notable increases in the PI_ABS_ (especially in CamBW1). In contrast, MD + F + GA reduced this parameter in LubBW1 and LubWa1.

For the traits observed at all six developmental stages, ANOVA was performed on data from DP_4 and DP_6 only for each genotype separately to analyze the interaction between treatment and DP. This interaction could be interpreted as a variable response to conditions in which plant regeneration could take place. The cases of significant interactions are shown in Table S5. The greatest number of significant interactions was recorded for CamBW1.

**Fig. 4:**
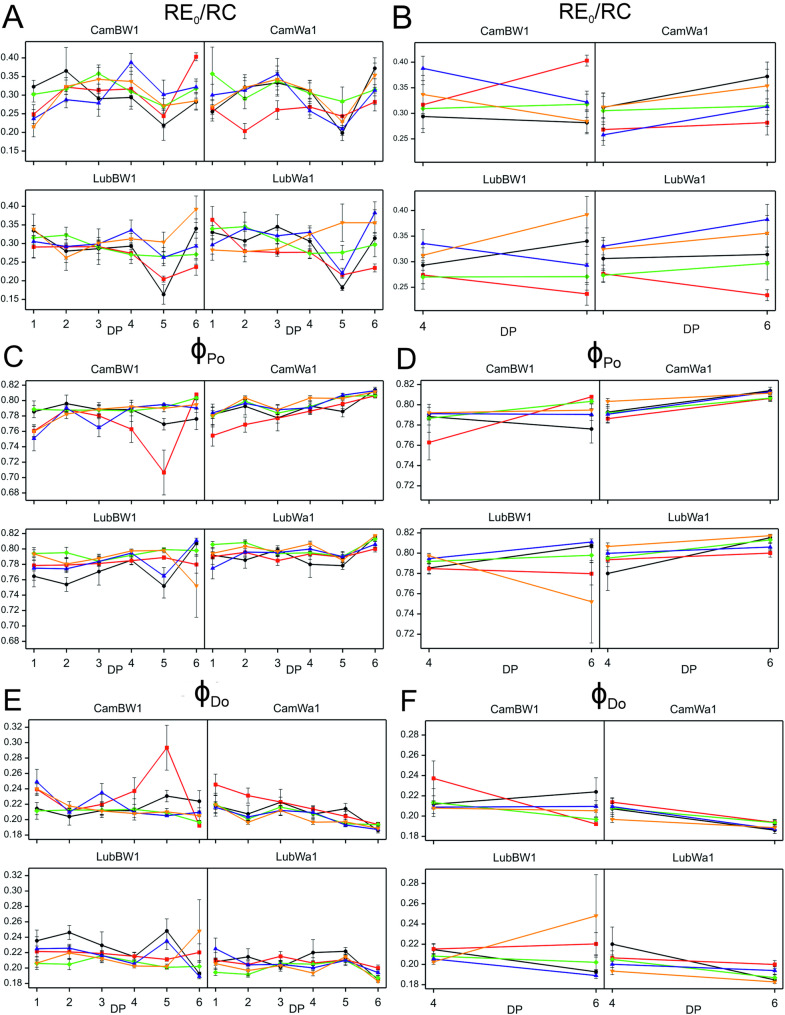
Variability in OJIP-derived parameters during drought and recovery in barley genotypes. The variability of the OJIP-derived parameter RE₀/RC is shown across all measurement points (DP_1–DP_6) (**A**) and at the cumulative drought duration time point (DP_4) and the end of the recovery period (DP_6) (**B**). The variability of the OJIP-derived parameter ϕ_Po_ is shown across all measurement points (DP_1–DP_6) (**C**) and at the cumulative drought duration time point (DP_4) and the end of the recovery period (DP_6) (**D**). The variability of the OJIP-derived parameter ϕ_Do_ is shown across all measurement points (DP_1–DP_6) (**E**) and at the cumulative drought duration time point (DP_4) and the end of the recovery period (DP_6) (**F**). Data (mean values with standard errors) are presented in arbitrary units. Treatments: black – control condition; red – MD + F + GA; green – MD + F + TR; purple – SD + F + GA; yellow – SD + F + TR. Parameter descriptions are provided in Table 1

For the CamBW1 genotype, a significant interaction was recorded for the following parameters: RE_0_/RC, ϕ_Po_, δ_Ro_, ϕ_Ro_, ϕ_Do_, PI_total_​. For the REo_RC parameter, a positive treatment effect was recorded at both DP_4 and DP_6 (Fig. 4). An increase in this parameter was observed during regeneration but only under MD conditions. The values of this parameter were also lower (compared with those of the control condition) at the onset of drought (DP_1) than at its peak (DP_4). Effective regeneration was observed only in MD.

**Fig. 5:**
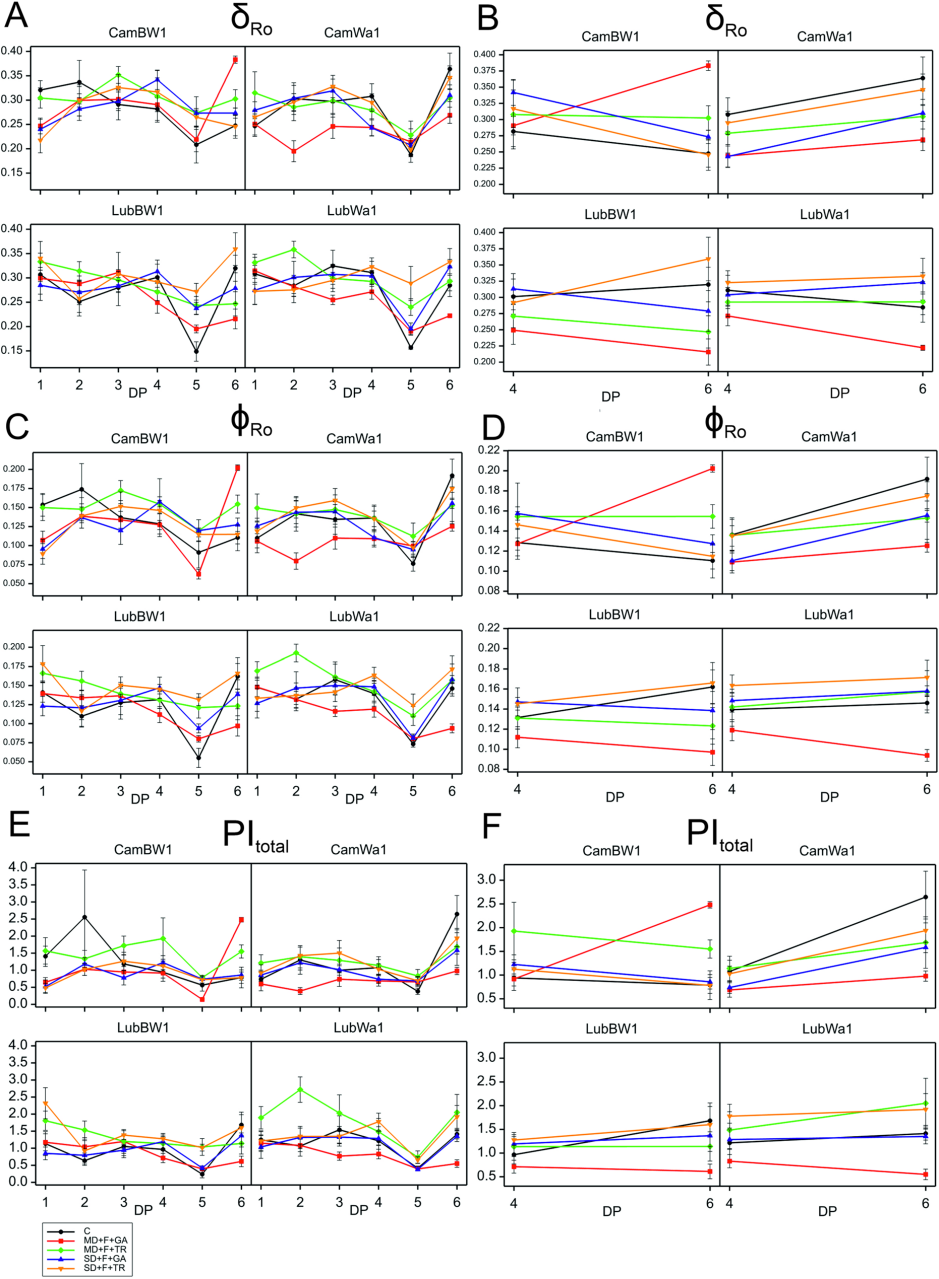
Variability in OJIP-derived parameters during drought and recovery in barley genotypes. The variability of the OJIP-derived parameter δ_Ro_ is shown across all measurement points (DP_1–DP_6) (**A**) and at the cumulative drought duration time point (DP_4) and the end of the recovery period (DP_6) (**B**). The variability of the OJIP-derived parameter ϕ_Ro_ is shown across all measurement points (DP_1–DP_6) (**C**) and at the cumulative drought duration time point (DP_4) and the end of the recovery period (DP_6) (**D**). The variability of the OJIP-derived parameter PI_total_ is shown across all measurement points (DP_1–DP_6) (**E**) and at the cumulative drought duration time point (DP_4) and the end of the recovery period (DP_6) (**F**). Data (mean values with standard errors) are presented in arbitrary units. Parameter descriptions are provided in Table 1

For the ϕ_Po_ parameter recorded for CamBW1, we observed positive treatment effects on DP_6—plants regenerated best under MD + F + GA conditions (Fig. 4 C, D). The average value of this parameter in this variant was the lowest at the peak of drought stress (DP_4). In the remaining variants—after initial fluctuations (DP_1–3) in the distribution of the curve plotted for ϕ_Po_ values—the parameter stabilized at levels similar to those recorded for plants growing under control conditions (DP_4). The values of this parameter were also lower in DP_1 than in DP_4 (an exception: MD + F + TR). For the ϕDo parameter, the plotted curves showed an opposite trend across the experimental variants—the average values of this parameter were relatively high in DP_4 under MD conditions but lower in DP_6. No significant changes in this parameter were observed under SD conditions between the drought period (DP_4) and the regeneration phase (DP_6). The values of this parameter were also greater at the onset of drought (DP_1) than at its peak (DP_4) (an exception: MD + F + TR). For the δ_Ro_ and ϕ_Ro_ parameters recorded for CamBW1, a similar distribution of plotted curves was observed (Fig. 5). The plants subjected to the MD + F + GA conditions regenerated best, with the highest mean values for the studied parameter (DP_6). Compared with those exposed to moderate stress conditions, plants subjected to SD conditions presented lower average δ_Ro_ and ϕ_Ro_ values at DP_6. Moreover, the values of these parameters in the plants subjected to MD conditions were greater (positive treatment effects) than those in the control plants under both DP_4 and DP_6. However, at the beginning of drought (DP_1), the values of these parameters were lower than those recorded for plants growing under optimal water conditions.

For the PI_tot_​_al_ parameter recorded for CamBW1, positive effects of the treatment were recorded, although it was still lower in DP_6 than in DP_4 (Fig. 5). However, under MD + F + GA conditions, at the peak of stress exposure, the average PI_total_​ parameter value was similar to that recorded for plants growing under control conditions. Moreover, an increase in PI_total_​ was significantly greater in DP_4 than in DP_6 (after regeneration). Lower values of this parameter (compared with the control condition) were observed in DP_1 than in DP_4 (except for MD + F + TR).

Delayed chlorophyll *a* fluorescence.

In this work, we focused on a few components of the recorded delayed fluorescence (DF) induction kinetics (I_1_, I_2_, D_2_) and the ratios between the maxima and minima of the induction curves (I_1_/I_2_) to better demonstrate the altered shape of the DF. Notably, for the CamBW1 genotype, higher I_1_ values were observed under MD conditions than under SD conditions (Fig. S9). For this genotype, the greatest *D*_2_ values were observed during the recovery stage in plants subjected to all types of applied stress conditions. Under control conditions, the lowest values of the ratio (I_1_ − D_2_)/D_2_ were observed for LubBW1 during DP_1, DP_2, and DP_5. The I_1_/I_2_ ratio was affected by the applied stress conditions. For example, under MD + F + TR, extremely high I_1_/I_2_ values were noted for CamBW1.

**Fig. 6:**
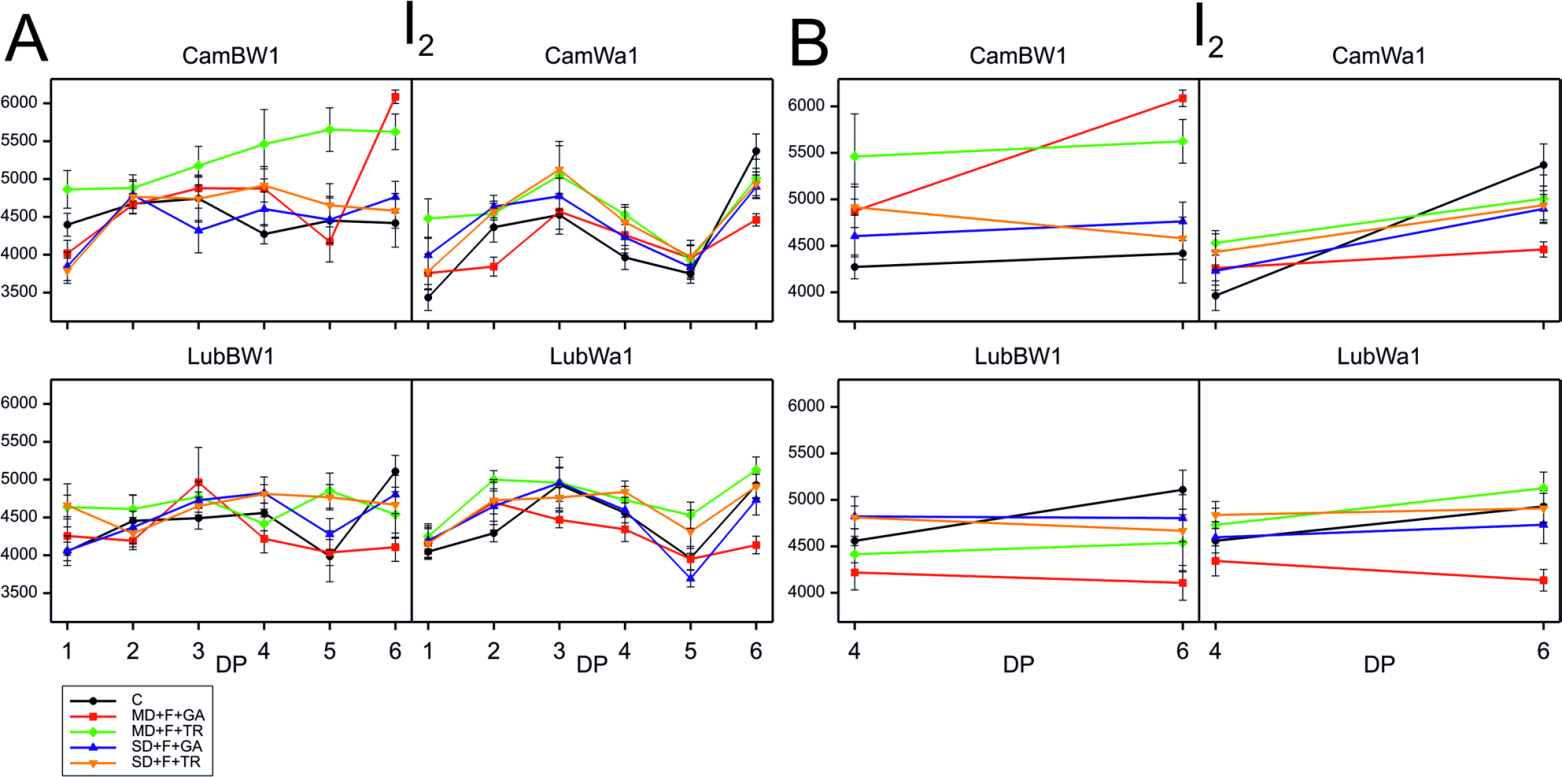
Curves of the delayed fluorescence induction of the barley genotype during drought and recovery. Delayed fluorescence induction curves across all measurement points (DP_1–DP_6) (**A**) and at the cumulative drought duration time point (DP_4) and at the end of the recovery period (DP_6) (**B**) for the parameters that significantly interacted in the studied genotypes grown under optimal water conditions and subjected to stress combinations. Data (mean values with standard errors) are presented in arbitrary units

Analysis of variance conducted on data from DP_4 and DP_6 to assess the interaction between treatment and developmental point revealed a significant interaction for the I_2_ parameter in the CamWa1 genotype (Table S5). For this parameter, positive effects were recorded for DP_4, and negative effects were recorded for DP_6 (Fig. 6). The plotted mean value curves for this parameter at the last drought phase and during regeneration show that the experimental variants with the same drought level (SD vs. MD) converge at similar points.

#### Modulated light reflection signal measured at 820 nm

In this study, no visible effects of genotype or treatment were observed for MR_0_. However, the transition at 820 nm was influenced by the applied stress conditions, as observed for the other studied parameters (Fig. S10). The highest MR_min_ values were recorded under control conditions for CamWa1. An increase in this parameter was observed in CamBW1, with a peak during DP_5 and a strong decrease in DP_6 under MD + F + GA conditions. A similar trend was observed under MD + F + TR but without a peak in DP_5. In MD + F + TR, the CamWa1 genotype exhibited a similar curve to that of CamBW1 under MD + F + GA. A disturbed course of the ∆MR_fast_ parameter curves under stress conditions was observed compared with that under control conditions. Under MD + F + GA, a strong decrease in ∆MR_fast_ was observed in DP_5 for CamBW1 and CamWa1 under MD + F + TR. A similar pattern was observed for the ∆MR_slow_ parameter in both CamBW1 and CamWa1. Additionally, a strong increase in ∆MR_slow_ was recorded in DP_5 under MD + F + TR for the LubWa1 genotype.

**Fig. 7:**
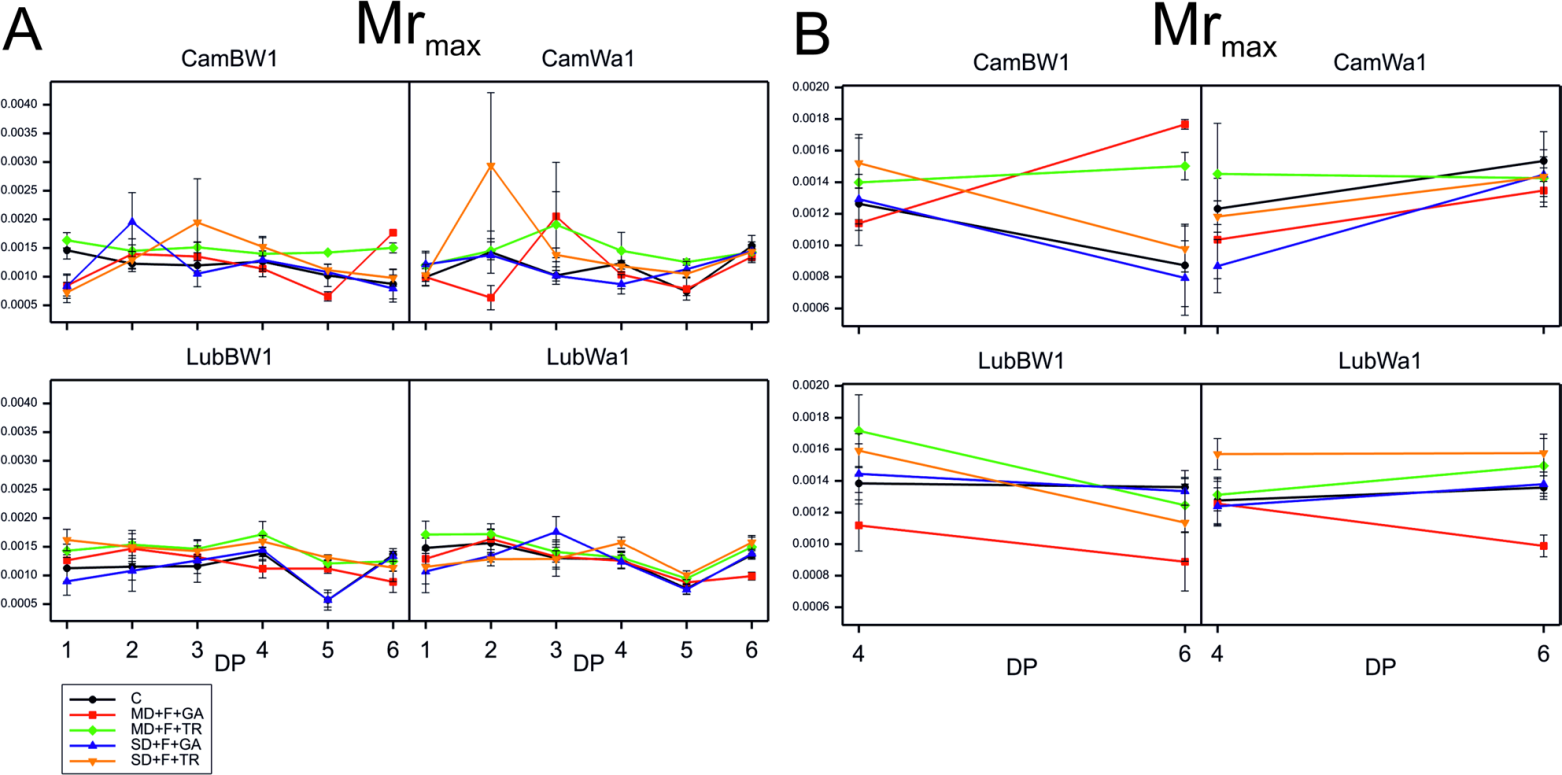
Kinetics of modulated light reflection at 820 nm during drought and recovery in barley genotypes. Kinetics of modulated light reflection at 820 nm across all measurement points (DP_1–DP_6) (**A**), at the cumulative drought duration time point (DP_4) and at the end of the recovery period (DP_6) (**B**), for the parameter that showed significant interactions in the studied genotypes grown under optimal water conditions and subjected to combined stress treatments. Data (mean values with standard errors) are presented in arbitrary units

Analysis of variance conducted on data from DP_4 and DP_6 to assess the interaction between treatment and developmental point revealed a significant interaction for the Mr_max_ parameter in **CamBW1**. At DP_4, the Mr_max_ curves were plotted close to each other due to the spraying treatment (GA and TR), whereas at DP_6, they were grouped on the basis of the type of drought (SD and MD) (Fig. 7).

### Chlorophyll content factor, nitrogen balance index and nondestructive flavonoid content measurement

The stress conditions affected the distributions of the Flav and Anth indices (Fig. S11). The value of Flav in the genotype CamBW1 in the nonstressed plants (C condition) was 1.4 units on the first measurement date, and no visible differences were found between the individual variants. For DP_2 in the variant SD + F + GA, the values of this parameter decreased. On the third measurement date, a decrease in the value of this parameter was observed in the MD + F + TR variant. On the DP_5 date, Flav levels decreased in all the variants, with the most pronounced reduction observed under severe drought conditions. For this genotype, a rapid increase in the Anth index value was also recorded in plants grown under the SD + F + GA treatment at DP_5. For CamWa1, the Flav curve for the SD + F + TR condition starts from the same point as the curve plotted for the control conditions and remains the highest during the drought period (DP_2–DP_4). For the LubBW1 genotype, the value of the discussed parameter was 1.2 units in plants that were not exposed to stress on the first measurement date, and an increase in the value of Flav was observed in the SD + F + TR, MD + F + GA, and MD + F + TR variants. On days DP_4 and DP_5, a decrease in this parameter was observed under all the applied conditions. On the last measurement date, the variants with severe drought presented lower Flav values than C—in contrast to the variants MD + F + GA and MD + F + TR. The value of Flav in the LubWa1 genotype was 1.4 units in DP_1, and the value of this parameter was found to be greater in the MD + F + GA variant. From DP_4 onward, only the values measured in the MD + F + TR variant presented higher values than those measured in nonstressed plants.

**Fig. 8:**
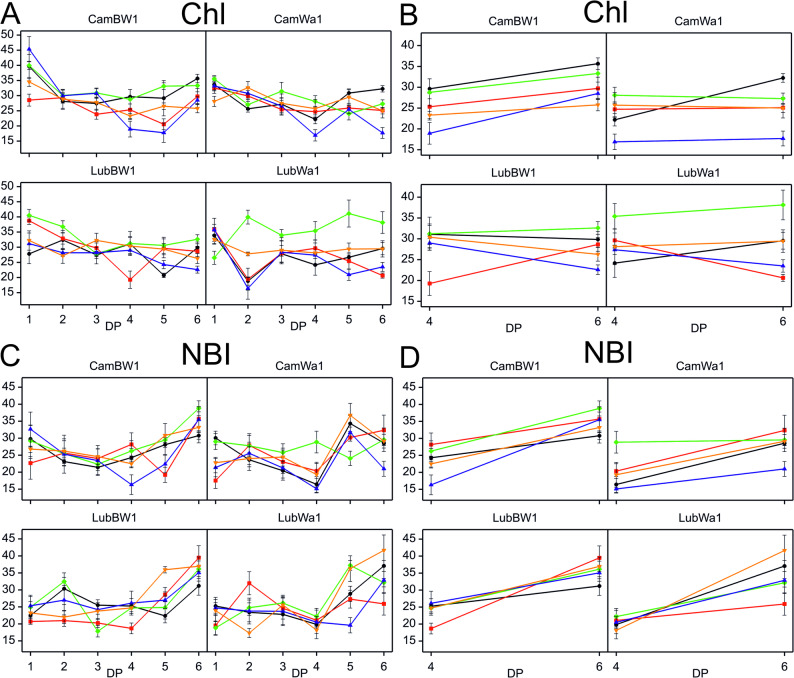
Variability of chlorophyll and nitrogen balance indices during drought and recovery in barley genotypes. The variability of the chlorophyll index (Chl) (**A**) and the nitrogen balance index (NBI) (**B**) is shown across all measurement points (DP_1–DP_6), as well as at the cumulative drought duration time point (DP_4) and the end of the recovery period (DP_6). Data (mean values with standard errors) are presented in arbitrary units. Treatments: black – control condition; red – MD + F + GA; green – MD + F + TR; purple – SD + F + GA; yellow – SD + F + TR

Analysis of variance conducted on data from DP_4 and DP_6 to assess the interaction between treatment and developmental point revealed a significant interaction for the Chl index parameter in the CamWa1 genotype (Table S5, Fig. 8). The Chl index values measured in plants growing under optimal moisture conditions increased over time (DP_4 to DP_6). The Chl index was greater in DP_4 than in the control for all the stress treatments (except for SD + F + GA). For the LubBW1 genotype, a significant interaction was recorded for the parameter Chl Index. The value of this parameter was reduced in DP_4 for all stress variants, except for similar Chl index values recorded under the C and MD + F + TR conditions. The increases were observed during the regeneration phase (DP_6) only for plants subjected to MD conditions. Notably, there was a significant increase in regeneration in the MD + F + GA variant, where a peak in this index value was observed in DP_6. However, the index values remained lower in this variant than in the control condition. For the LubWa1 genotype, a significant interaction was recorded only for the NBI index. For this parameter, similar mean values were recorded across different conditions at DP_4. However, during the regeneration phase (DP_6), greater variation in NBI values was observed across different stress variants. Only the SD + F + TR variant exhibited a positive treatment effect.

### Evaluation of yield-related traits

The results of the analysis of variance (ANOVA) revealed significant effects of the applied treatments on almost all the investigated yield-related traits (except for PTn and TGW). On the other hand, no significant effects of genotype were found for these traits in this experiment (Table S4).

A significant increase in the mean Tn value under stress conditions was observed for almost all the studied genotypes (with the exception of CamWa1) (Fig. S12). Notably, significant increases in the mean PTn values were recorded only for LubBW1 under mild stress conditions. A negative impact of the applied stress conditions on the grain yield was observed in this study, although some changes in the GY mean values were not significant. The largest decrease in the GY mean was observed for LubWa1 plants grown under severe drought conditions. For the traits related to the spike morphology of the studied plants, a decrease in the mean values was observed under all stress conditions compared with the optimum water conditions. The spike morphology of LubWa1 was particularly negatively affected by stress conditions—relatively low mean values of the traits LSm, NSSm, NGSm, and WGSm were observed for this genotype, especially in plants subjected to severe stress (Fig. S13). On the other hand, lower NSSm, NGSm, and WGSm mean values were observed for the genotype CamBW1 under stress conditions associated with external GA application than under the stress conditions under which the second growth regulator was applied.

For all the traits related to the lateral spike morphology of the plants subjected to stress conditions, lower mean values were observed in the studied genotypes than in the control conditions (Fig. S14). Remarkably, stimulating effects (although, in some cases, not significant) (compared with mild drought conditions) of severe drought in combination with GA application on LSl, NSSl, NGSl, and WGSl were observed for CamBW1 and CamWa1.

### Expression analysis of WRKY transcription factor genes

During the experiment, we can distinguish two experimental periods: stress (DP_1–DP_4) and recovery (DP_5–DP_6). Analysis of variance conducted on data from DP_4 and DP_6 to assess the interaction between treatment and developmental point revealed a significant interaction for all three studied genes (Table S5) at two developmental points, DP_4 and DP_6.

**Fig. 9:**
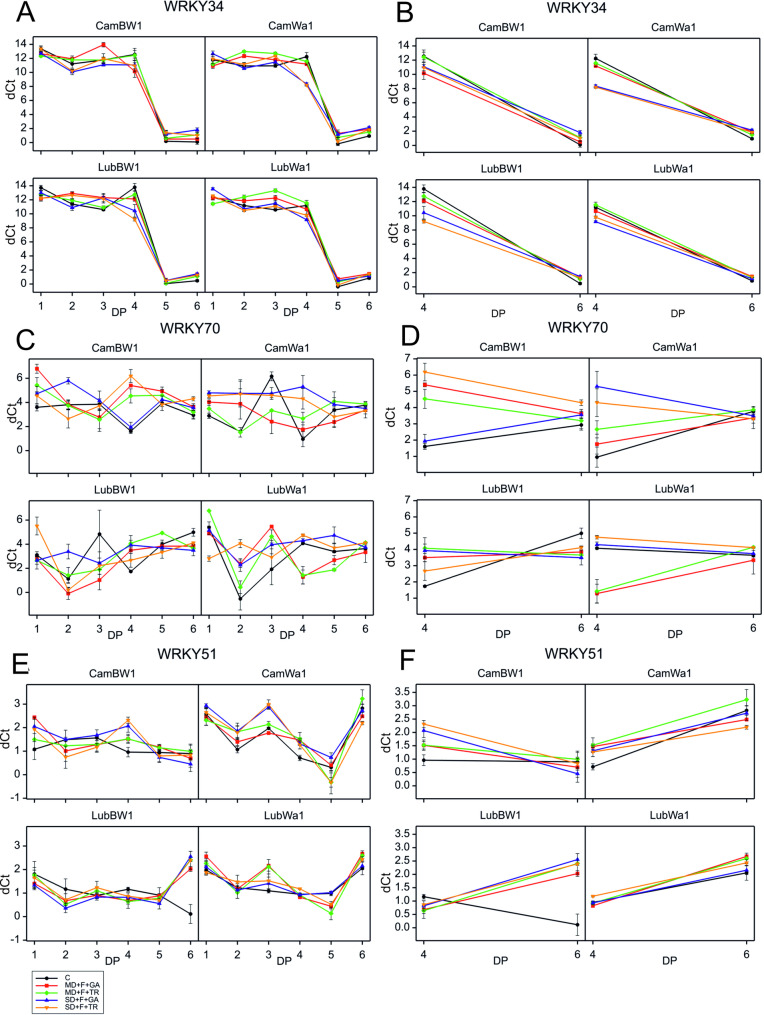
Expression patterns of WRKY transcription factors during drought and recovery in barley genotypes. Analysis of the expression patterns of the studied transcription factors across all measurement points (DP_1–DP_6), at the cumulative drought duration time point (DP_4) and at the end of the recovery period (DP_6) (**B**), in plant tissues collected from barley genotypes differing in phenology for WRKY34 (**A**, **B**), WRKY70 (**C**, **D**), and WRKY51 (**E**, **F**).

The expression of WRKY34 followed a similar pattern across all the experimental variants: it increased upon stress application (Fig. 9); however, under DP_4, it was still lower than the expression observed in the control variant. Notably, at DP_3, we noted higher expression levels in almost all the variants than at DP_4 (except for MD + F + TR in CamBW1); hence, there were negative treatment effects at this time point. After the plants were restored to control conditions, a sharp decrease in expression levels was observed, but over time, at DP_6, WRKY34 expression increased again, and we also observed positive treatment effects—variants exposed to stress maintained a greater expression level than did plants that grew continuously under optimal conditions. Under the MD + F + GA condition, a rapid increase in the WRKY34 expression level was recorded at DP_3 for CamBW1. No such changes were observed under mild drought conditions combined with TR application. In general, differences between genotypes are not large—all groups show the same increase in WRKY34 expression (DP_5 → DP_6).

The expression pattern of *WRKY70* varied among the genotypes as well as under the applied conditions (Fig. 8). Developmental progression does not appear to have an influence—the expression curve observed in the control does not reflect those recorded under controlled conditions. Positive treatment effects were observed for CamBW1 at both development points (DP_4 and DP_6), whereas for LubBW1, positive treatment effects were recorded at DP_4, and negative treatment effects were recorded at DP_6. A high expression level was noted at DP_4, during the peak of stress impact. In most cases, expression is high at DP_1, but as stress factors take effect, it decreases until DP_3, followed by an increase at DP_4, leading to positive treatment effects at DP_4 (with the exception of variants with MD conditions in LubWa1). We observed significant differences in WRKY51 expression between the peak of drought stress (DP_4) and the recovery period (DP_6), but only in two examined objects: CamBW1 and LubWa1 (Fig. 8). The expression pattern for both studied genotypes resembles that of WRKY70; it is high at DP_1 for stress variants, followed by a decline and a peak at DP_3 for LubWa1 and DP_4 for CamBW1 in each stress variant.

## Discussion

### Plant phenology and trichome micromorphology

In this study, we decide to employ a combination of extremely early-heading Syrian breeding line Cam/B1/CI08887//CI05761, the late-heading Polish cultivar Lubuski, and genotypes with strong wax development on the leaf and spike surface (Keops), as well as the glossy Bowman backcross-derived line BW408. Eceriferum Bowman backcross-derived lines are specifically designed to study the genetic basis of wax production and trichome development in barley plants [42]. This approach allowed us to explore, among other factors, the role of the epidermal structure in the multifactorial stress response of barley plants with different phenologies.

Our study revealed significant genotype-dependent differences in phenology, *HvPRR37* expression patterns, and trichome density and morphology under stress conditions, including distinct variation in the timing of key developmental stages such as flag leaf emergence, flowering, and heading. Generally, genotypes with late-heading progenitors (such as Lubuski) reach the tillering stage later than those of Syrian origin (such as CamBW1 and CamWa1), which is in line with observations from previous investigations [28, 43]. Under drought stress conditions, the number of days to flowering significantly increased, particularly for early-heading plants subjected to severe drought. In contrast, late-heading plants presented consistent values under both mild and severe drought conditions. These results confirm our previous conclusion about the role of the “drought escape” (DE) strategy adapted by early-heading barley genotypes [43] and highlight the fact that this resistance mechanism can be efficient only when plants complete the vegetation period before the environmental conditions become unfavorable.

In our study, a molecular approach was also employed to confirm phenological differentiation among plants, revealing that although *HvPRR37* expression patterns were generally similar, their expression levels varied across genotypes. Notably, all the studied genotypes experienced a rapid increase in *HvPRR37* expression postflowering, with the highest peaks observed for LubBW1. In barley, a long-day (LD) crop, the PSEUDO-RESPONSE REGULATOR (*HvPRR37*) gene, also known as PHOTOPERIOD RESPONSE LOCUS1 (*Ppd-H1*), is the central heading time gene regulated in response to LDs, whereas recessive alleles (*ppd-H1*) reduce the response to LDs [44].

Although no statistically significant differences in trichome density were found among the genotypes grown under optimal water conditions, subtle variations in trichome micromorphology were nevertheless observed through the analysis of scanning electron microscopy (SEM) images. LubBW1 presented the highest values for certain morphological traits, such as trichome area, whereas LubWa1 presented the lowest values for these traits. Conversely, traits related to trichome micromorphology presented the lowest values for LubBW1, suggesting the existence of potential variability in disease resistance associated with these morphological characteristics. In other words, the SEM observations revealed that both cross combinations with the Polish ancestor (Lubuski) were characterized by opposite trichome micromorphology—LubBW1 had many tiny trichome structures, whereas LubWa1 developed fewer large trichrome structures. Although trichomes are traditionally known for their role in physical defense against herbivores and environmental stress, they also contribute to the plant’s defense against pathogens such as fungi [45]. There are reports describing the characterization of the trichome structure as ‘an anchor’—a sticky point that enables the spore to colonize plant tissues [46]. On the other hand, trichomes can create a physical barrier that can limit the access of fungal spores to the epidermis of barley leaves or stems. The presence of these structures makes it more difficult for fungi to reach and penetrate a plant’s surface, delaying or preventing infection in plants [47]. In certain cases, trichomes can trap fungal spores or limit their access to stomata, which are common entry points for fungal pathogens. By covering or shielding stomata, trichomes reduce the chances of fungal penetration into plant tissues [48], a phenomenon that was most likely noticed during this study *via* SEM observations.

### *Fusarium*-induced effect evaluation

While direct comparisons of severity were generally not statistically significant in our study, the plant responses suggest that LubBW1 and LubWa1, which are genotypes with many tiny trichome structures and a small number of large trichome structures, respectively, may exhibit different susceptibilities under certain stress conditions. Furthermore, the results of the analysis revealed that the genotypes did not significantly differ in the severity of *Fusarium* infection on the basis of the fungal biomass, percentage of infected spikes, or DON levels, suggesting that while visual symptoms vary, the underlying resistance may not differ significantly across the studied genotypes. We also observed a much greater level of visual evaluation (as well as DON level) in LubWa1 than in LubBW1, which may suggest that trichome localization in the inner part of LubBW1 plant cuffs can act as a trap for fungal spores; these results are in line with the data shown in Imboden et al. [14]. In our experiment, we observed many trichome traps surrounding the stomata, with “captured” *Fusarium* conidia, but this function is presumably closely related to the morphology of the trichomes.

In our study, the results of ANOVA revealed significant effects of applied stress conditions on the number of trichome structures—increases in trait trichome density in plants grown under severe drought combined with *Fusarium* infection and GA application were detected (with the exception of CamBW1). Studies have shown that gibberellin (GA) [49, 50] and other phytohormones [51, 52] regulate the initiation, growth, and development of plant trichomes. GA treatment increases trichome numbers on Arabidopsis leaves [53]. Mild drought generally leads to a slight decrease in gibberellin levels, resulting in a reduction in the growth rate but not complete cessation [54], but severe drought causes a significant reduction in gibberellin biosynthesis. This may explain why we did not observe a similar effect in the SD variant combined with TR application. Lower GA levels inhibit cell elongation and seed germination, effectively halting growth to conserve resources. This is a survival mechanism in which the plant focuses on enduring stress rather than growing [55]. When plants face drought conditions, they undergo complex hormonal changes that help them adapt and survive [56]. Drought severity can lead to distinct hormonal shifts, altering the production, signaling, and interaction of key hormones such as abscisic acid (ABA), ethylene, auxins, gibberellins, cytokinins, jasmonic acid, and salicylic acid [57, 58]. These findings explain the variations in fungal infection responses of plants subjected to two types of drought (as well as the application of exogenous GA) in our study.

### Photosynthetic performance of plants

Analysis of chlorophyll fluorescence (ChlF) induction curves allows evaluation of the physiological conditions of photosystem II (PSII) and photosynthetic electron transport chain components [17]. This technique provides quantifiable data that can be used to compare the severity of stress across different plants or treatments [59]. The multiparametric approach provides more complex information and may provide a better view of the plant’s responses to stress, which can be elusive, especially with respect to the dynamic changes in plant development [60]. Therefore, in our study, different tools were used to estimate plant photosynthetic performance under stressed conditions. Moreover, as suggested by Dąbrowskie et al. [32], simultaneous measurements of PF, DF, and MR_820_ signals can be used to accurately determine electron transport through PSII and PSI. Thus, it can provide broader and more detailed information on structural and functional changes in PSII and PSI under a combination of abiotic and biotic stresses.

The results of the analysis revealed that a combination of abiotic and biotic stresses had a significant effect on the efficiency of the photosynthetic apparatus of spring barley plants and that this response also depends on the genotype and the time of measurement (DPs). These results are in line with previous studies reporting that the mechanism of photosynthesis is strongly affected under different single abiotic stresses [61, 62] as well as under multiple stress combinations [63] by modulating photosystem I (PSI)- and photosystem II (PSII)-mediated chemical reactions and chlorophyll biosynthesis [64]. The analysis of the OJIP curves revealed that the SD + F + GA condition impaired the function of photosystem II (PS) from the second measurement time point (DP_2); the curves drawn for the CamBW1 and CamWa1 genotypes decreased at points I and P, and the distribution of the curve drawn for the LubWa1 genotype decreased at points J and P. These findings can be explained by the natural resilience of the parent (CamB) used for obtaining these cross combinations to drought and the effect of the stress duration period on the plant [43]. At the last date of the stress application, the most negative effect was caused by the SD + F + GA conditions for all the varieties (with the exception of LubBW1). Notably, mild drought (MD) caused relatively small changes in the OJIP curves. Drought can have significant effects on plants, depending on their severity and adaptability [65]. Mild drought stress causes plants to conserve resources and limit growth, whereas severe drought can lead to irreversible damage and plant death if the conditions persist [65, 66]. Under mild drought, plants experience moderate water stress but can still perform photosynthesis at a reduced rate. However, various physiological adjustments occur to cope with the limited water supply, which we believe took place in our study’s case of the “behavior” of certain genotypes.

In our study, the analysis of the treatment × development point interaction at two selected points (DP_4 and DP_6) revealed a significant interaction, among others: ϕ_Po_, δ_Ro_, ϕ_Ro_, ϕ_Do_, and PI_total_​ only for one studied genotype: CamBW1. The study described these parameters as they collectively provide insight into how the treatment affects the photosynthetic machinery at different developmental stages. The significant interaction at DP_4 and DP_6 indicates that the treatment influenced plant physiological responses in a developmentally dependent manner, leading to differences in photosynthetic efficiency, energy dissipation, and electron transport dynamics over time.

For the PI_total_ parameter, positive effects of the treatment were recorded; however, they were still lower in DP_6 than in DP_4 (with the exception of the MD + F + GA condition). This is particularly intriguing because the parameter PI_total_ (total performance index), as the key indicator of the overall efficiency and vitality of the photosynthetic machinery in plants, is related to photosystem II (PSII) and energy conversion processes [67]. The overall sensitivity and ability to recognize the differences between the drought-sensitive and drought-tolerant genotypes were highest for PI_total_ in many previous studies [60, 68], thus confirming the exceptional suitability of this indicator for determining drought stress effects and screening genotypes according to their drought tolerance. Therefore, we expected lower average values of this parameter to be recorded at the peak of drought stress (DP_4). However, analyzing all six points of the curve indicates that the plants adapted to unfavorable conditions, most likely by activating defense mechanisms such as osmolyte accumulation or efficient stomatal closure [69]. Moreover, under MD + F + GA conditions, for the CamBW1 genotype, the average values of the PI_total_ parameter at the peak of stress exposure were similar to those recorded for plants growing under control conditions. Interestingly, the applied stress even increased the PI_total_ parameter, as it was significantly greater than that in DP_6 (after regeneration). This phenomenon can be explained by the previously described impact of different drought levels on plants (severe drought versus mild drought) and by the application of gibberellin (GA) to these plants. Gibberellins promote cell elongation and shoot growth [70] while enhancing photosynthesis and activating related genes under stress conditions [71, 72]. Hence, similar values of the PI_total_ parameter under control conditions and MD + F + GA may not be surprising. Interestingly, lower values of this parameter were observed in DP_1 than in DP_4 (except for MD + F + TR), indicating that this genotype has a flexible ability to adapt to unfavorable environmental conditions.

The ϕP₀ parameter reflects the efficiency of light energy transfer within PSII [73], which typically decreases under drought conditions as reduced water availability and ROS accumulation in thylakoids can damage PSII reaction centers [74, 75]. For this parameter, we recorded positive treatment effects in DP_6—indicating that the plants had regenerated—most notably under MD conditions. Under severe drought stress conditions and MD + F + TR, the decrease in ϕ_P0_ was minor, as the examined plants could compensate for stress effects, for example, by regulating stomatal function [76]. We did not observe these patterns for the plants subjected to MD + F + GA. Interestingly, the average values of this parameter in the variant above were the lowest during the peak of drought duration; the moderate drought level combined with GA application did not sufficiently stimulate the plants to switch to a “defense mode.” Under severe drought stress, a significant decrease in this parameter is typically observed, which may indicate permanent PSII damage and a transition of the plant into a state of chronic stress [77]. However, such a phenomenon was not observed in the studied genotype (an exception: MD + F + GA). Indeed, in the initial phases of stress, a decrease in the average value of this parameter was recorded, but over time (DP_4), the studied genotype presumably activated defense or stress-mitigation mechanisms. Consequently, at the peak of drought stress, we observed similar values across almost all stress variants to those recorded for the control group.

The ϕ_Ro_ and δ_Ro_ parameters, reflecting photosystem I activity and the final stage of electron transport, are specifically affected by drought, which disrupts the entire photosynthetic electron transport chain, including the PSI-dependent phase [78, 79]. The high values of ϕ_Ro_ and δ_Ro_ indicate that more electrons have reached PSI to reduce final electron acceptors at the PSI acceptor side, which could signify an over compensatory effect on quantum yields and efficiency at the PSI acceptor side [80]. The plants subjected to the conditions applied in MD + F + GA regenerated best, as the highest average values were recorded for the studied parameters. In plants subjected to SD conditions, lower average values of δ_Ro_ and ϕ_Ro_ were recorded than those in plants exposed to moderate stress conditions, which highlights the importance of the intensity (as well as duration) of stress conditions. Concurrently, the values of these parameters were greater under stress conditions than under control conditions for both DP_4 and DP_6, which may be related to the adaptive ability of the CamBW1 genotype. In DP_1–DP_2, the values of these parameters were lower—only in DP_3 or DP_4 did their values increase, possibly due to the activation of alternative electron transport pathways in plants, such as cyclic transport around PSI [81].

For the parameter ϕ_Do_, we observed an opposite trend in the curves (compared with δ_Ro_ and ϕ_Ro_) plotted for the applied experimental variants—the average values of this parameter were relatively high in DP_4 under MD conditions. In contrast, lower values were observed in DP_6. Since drought reduces PSII efficiency (ϕ_Po_) and ϕ_Do_ has the opposite effect, increasing this parameter is a natural consequence of water stress [82]. When PSII is less efficient, the plant dissipates more energy as heat and fluorescence to avoid damage to the photosynthetic system. The values of this parameter were also greater at the beginning of drought application—DP_1—than at its peak (DP_4). The high values of this parameter in plants subjected to MD + F + GA conditions can be explained by the fact that the studied plants may have actively increased ϕ_Do_ through thermal energy dissipation (nonphotochemical quenching, NPQ) [83] to prevent PSII damage.

The REo/RC parameter determines how many electrons are transferred to PSI per PSII reaction center [84]. A positive treatment effect was recorded for this parameter at both DP_4 and DP_6. However, the values of this parameter were also lower (compared with those under the C condition) at the beginning of drought application—DP_1—than at its peak (DP_4), suggesting (a relatively rapid—DP_3) adaptation of plants to stress conditions. An increase in the mean values of this parameter during the recovery phase was recorded only for MD conditions, indicating that plants regenerated less effectively under severe drought conditions.

The modulated reflectance signal (MR) is measured at 820 nm, and it provides information about electron transport adjacent to plastoquinone (PQ) and to PSI acceptors [32, 85]. PSI can become excessively reduced or damaged under stress conditions (e.g., drought, high light, or nutrient deficiency). The parameter MR_max_ refers to the maximum value of the modulated light reflection signal at 820 nm, which is related to the dynamics of oxidation and reduction of the reaction centers of photosystem I (PSI), especially the oxidative state of P700 and plastocyanin (PC) [32]. A decrease in electron availability leads to a lower amplitude of signal change at 820 nm, thereby reducing MR_max_. On the other hand, if PSI cannot accept electrons, excessive oxidation of PSI efficiently may occur, increasing MR_max_. Drought-resistant plants often exhibit relatively stable values of this parameter [86]. Hence, in this study, a positive treatment effect for the MR_max_ parameter was recorded in DP_6 for variants under MD conditions (small positive effects were also noted for SD + F + TR). Additionally, after the initial fluctuations (DP_1–DP_3) in the distribution of curves plotted for this parameter, at the peak of the drought (DP_4), CamBW1 plants exhibited relative stability of this parameter (values similar to those recorded for plants grown under the C condition). It appears that CamBW1 plants are capable of activating efficient defense mechanisms under severe drought conditions.

For the CamWa1 genotype, a significant interaction was recorded for the I₂ parameter and the Chl index. The I₂ phase of DF is highly sensitive to environmental stressors and treatments [87], with changes in this parameter reflecting alterations in PSII electron transport capacity and photoprotection mechanisms. Since, in this study, we recorded significance in terms of treatment × development point interactions for the parameter I₂, the results suggest that the treatment affected PSII charge recombination and energy dissipation differently at DP_4 and DP_6. Interestingly, positive effects for this parameter were recorded in DP_4, whereas negative effects were observed in DP_6. This may suggest that despite adapting CamWa1 plants to drought conditions, they could not recover to the extent observed in the control plants.

Stress factors can reduce chlorophyll content in plants, leading to chloroplast damage, decreased photosynthetic efficiency, and premature leaf senescence, ultimately diminishing overall plant vigor [88]. Biotic stresses such as those caused by pathogens (fungi) can lead to a reduction in chlorophyll content and tissue damage or disruption of plant metabolic processes [89–91]. The average values of the Chl index measured in plants growing under optimal moisture conditions increased over time (DP_4–DP_6), which is consistent with the literature [92, 93]. Interestingly, the Chl index was greater in DP_4 than in the control for all the stress treatments (except for SD + F + GA). This index also exhibited a certain degree of stability—plants that experienced stress did not increase in value as much as those in control conditions did. A possible explanation for this is compensatory mechanisms in response to stress. Plants may gradually increase their chlorophyll synthesis or chloroplast density to increase their photosynthetic efficiency and maintain metabolism in the presence of limited resources [94, 95]. In some cases, stress (e.g., moderate drought) may cause a temporary increase in chlorophyll accumulation as an adaptive mechanism before a long-term decline in photosynthetic activity. The SD + F + GA variant may have exhibited different response mechanisms, most likely related to the effects of gibberellin.

For the LubBW1 genotype, two parameters (the Chl and NBI indices) showed a significant interaction. The Chl index was reduced in DP_4 for all stress variants, indicating entirely different mechanisms activated by LubBW1 plants compared with the CamWa1 genotype. An increase during the recovery phase (DP_6) was observed only in plants subjected to MD conditions. Notably, plants exposed to SD conditions did not show an increasing trend in this index after recovery.

For the LubWa1 genotype, only one parameter (the NBI index) showed a significant interaction. When plants are subjected to stress, their metabolism shifts between growth (requiring nitrogen) and defense responses (which involve flavonoid production) [96]. Drought limits the availability of nitrogen because reduced water availability impairs nutrient uptake. Moreover, drought triggers the production of antioxidants and flavonoids to protect plants from oxidative damage [97]. For the NBI index, similar average values were recorded at DP_4; however, during the recovery phase (DP_6), more diverse NBI values were observed across different stress variants. The studied plants of this genotype regenerated differently depending on the type of applied stress. In general, the severity of stress determines the availability of resources for recovery, the activation of various hormones, and the level of tissue damage [98], which was clearly evident in the present study.

### Evaluation of yield-related traits

Significant effects of the applied treatments on various yield-related traits in the studied genotypes were observed in this study. However, no significant effects of genotype were observed, which pinpointed some level of morphological similarity between the studied plants. The analysis indicated that all the tested genotypes experienced a reduction in yield-related traits under stress conditions; however, the magnitude of this reduction varied. Additionally, the significant impacts on traits such as the number of spikes, spike length, and yield suggested that environmental stressors (such as drought and *Fusarium* infection) play a substantial role in yield performance, which has been reported for different crops, including wheat [99] and barley [100]. Interestingly, in plants subjected to stress conditions, relatively high Tn mean values were probably associated with the development of secondary tillers—a process that we also reported in our previous experiments [28, 29, 43]. Severe drought conditions caused a rapid decrease in the number of productive tillers in almost all the studied genotypes, with one exception—CamBW1—which presented a high PTn mean value, but the quality of the spike yield was very low. This finding shows that this genotype represents a Syrian survival strategy—the development of tillers with fertile spikes (but of low quality/with only a few grains) was the main goal of the plant’s strategy to survive (to distribute the progeny) under unfavorable conditions. This finding is in line with other studies [101, 102] and with the results from our previous experiments [43, 103].

### Expression analysis of WRKY transcription factor genes

WRKY transcription factors are a large family of plant-specific regulators that modulate stress-responsive gene expression and play key roles in plant development, metabolism, hormone signaling, trichome morphogenesis, and defense against pathogens [104–106].

In our study, we selected three different TFs from two different subgroups, as described by Cheng et al. [107], to demonstrate the variability in the expression patterns of these TFs in the context of multiple stresses. While specific studies on WRKY34 in barley might be limited, insights can be drawn from general knowledge about WRKY transcription factors and related research in barley and other plants. Each homolog of the WRKY genes presented the closest similarity, such as AtWRKY53 with TcWRKY53 or AtWRKY70, BrWRKY70, and MfWRKY70 with TaWRKY70. On the other hand, AtWRKY53 expression is induced by drought stress [108]. In contrast, the expression of TcWRKY53 is induced by cold stress [109], indicating that these two WRKY genes play different roles under different abiotic stresses and in different species. It was also assumed that each WRKY gene might also contribute to multiple abiotic stresses. The overexpression of GhWRKY34 and GhWRKY41 reportedly increases the salt tolerance of *A. thaliana* and the drought tolerance of tobacco [110]. In our study, the WRKY34 expression level in most cases increased upon stress application, which, on the one hand, suggests its role in the stress response. Compared with the control plants, the stressed plants exposed to stress presented greater WRKY34 expression even after the stress ended. This may indicate “stress memory” and its influence on adaptive mechanisms [111]. On the other hand, a similar expression pattern was observed in the control condition, suggesting that WRKY34 is not solely responsible for the stress response but may also play a role in developmental processes, although further studies are needed to confirm this. This finding aligns with studies describing the role of this transcription factor in the development of various plant structures. For example, recent research has identified two particular TFs, WRKY34 and WRKY2, that are involved in the development of male gametes in *A. thaliana* [112]. Over time (DP_6), the expression level of this gene increases again, indicating its possible role in later stages of plant regeneration or development. While some authors [113] suggest a connection between this factor and fungal infection by *Fusarium* species, our results clearly indicate a strong association of this gene with plant development.

The group III WRKY proteins, which include both WRKY51 and WRKY70, are believed to play important roles in the plant response to stresses [114]. The WRKY transcription factors (TFs) WRKY46, WRKY54, and WRKY70 in Arabidopsis are linked to the response of plants to drought and their development, which is mediated by brassinosteroids (BRs). These hormones play crucial roles in regulating the growth of plants, including spring barley [115]. The mutants (wrky46, wrky54, and wrky70) of these TFs presented modified plant development and controlled expression of genes involved in the drought response [116]. The initial stage of wheat streak rust infection is also characterized by a significant increase in the TaWRKY70 expression level at elevated temperatures. This finding reflects the positive correlation between TaWRKY70 expression and the resistance of wheat seedlings to heat stress as well as the likely activation of the SA and ET signaling pathways during the initial stage of infection [117]. The TaWRKY70 gene was confirmed to be located within the QTL-2DL region and imparted resistance against FHB [118]. In the case of the WRKY70 expression curves, developmental progression does not appear to have an influence—the expression curve observed in the control does not reflect those recorded under controlled conditions. A high expression level was noted at DP_4, during the peak stress impact for almost all the genotypes and applied treatments (except for LubWa1 under MD conditions). Since the effects of treatment are diverse in DP_4 but similar in DP_6, this implies a potential link between the expression of this gene and the response to the applied stress variants. As the roles of WRKY70 in fungal pathogens have been described [106], we also observed significant fluctuations in the expression curve in response to a combination of abiotic and biotic stresses. In most cases, expression was high at DP_1, but as stress factors took effect, it declined until DP_3, followed by an increase at DP_4, leading to positive treatment effects at DP_4 (except for variants with TR application in LubWa1). This may indicate that the expression initially increased in response to *Fusarium* infection (first infection treatment – DP_1), but as drought stress progressed, it was suppressed and then increased again after the second infection treatment (DP_3). Thus, a link between WRKY70 expression levels and infection can be inferred, while this response appears to be short-lived.

In our study, for CamBW1 plants grown under the control condition, the WRKY51 expression level remained stable throughout the experiment, whereas for all stress variants (especially severe drought), it remained high (DP_4), after which we observed a decrease in expression during recovery. The analysis of the expression curve for LubWa1 revealed the appearance of the expression peak much earlier (DP_3), which may indicate the activation of a similar resistance mechanism on the basis of the activation of WRKY51 expression by LubWa1 but at a different developmental stage; the question of whether this fluctuation was related to the duration of drought or the susceptibility of this genotype to stress remains open.

The findings of this study have broader implications for barley improvement under drought-prone conditions. The observed relationships between phenology, trichome micromorphology, and the regulation of WRKY transcription factors highlight key traits that may contribute to stress tolerance. In particular, early-heading genotypes employing a “drought escape” strategy, together with specific epidermal features such as dense, fine trichomes, may serve as valuable targets in breeding programs aimed at enhancing drought and pathogen resilience. Moreover, the identification of WRKY51 and WRKY70 as stress-responsive genes reflects their potential as molecular markers for selecting tolerant genotypes. Future studies should focus on validating these candidate genes under field conditions, exploring their regulatory networks, and integrating phenological and epidermal traits into multi-trait selection models for drought-tolerant barley breeding.

## Conclusions

The main objective of this study was to analyze the expression dynamics of selected transcription factors in barley plants exposed to combined abiotic and biotic stress - our findings indicate that varying stress intensities, specifically mild and severe drought conditions, differentially affect the expression levels of the studied transcription factors. These results confirm that WRKY TF expression is rapid, transient, and tissue specific. Additionally, we demonstrated that the expression levels of the genes WRKY51 and WRKY70 responded significantly to stress (or its intensity), whereas the expression level of the gene WRKY34 was dependent on plant development. We also demonstrated a relationship between WRKY70 expression levels and infection; however, we also found that this response was temporary.

The simultaneous analyses of the JIP test, DF, and MR data revealed that the applied stress conditions had a negative effect on the efficiency of the photosynthetic apparatus. Notably, mild drought caused smaller changes in the OJIP curves. The current efficiency of photosystem II was inhibited to a lesser extent by the given stress factors than was the potential efficiency of photosystem II. The combination of severe drought, *Fusarium*, and GA also had the greatest negative effect on the chlorophyll content (greenness index) in the leaves of all the studied genotypes. Photosystem I (PSI) was also inhibited by the impact of stress factors, as evidenced by changes in the MR_820_ curves. This part of the photosynthetic apparatus was also inhibited to the greatest extent by severe drought, *Fusarium*, and GA application in all four studied genotypes. The genotype least affected by all stress factors was the LubBW1 genotype.

Unfortunately, owing to the lack of statistically significant differences, we cannot conclusively determine whether certain genotypes are more or less resistant to *Fusarium* fungal infections. However, we were able to characterize the trichome morphology of two genotypes, LubBW1 and LubWa1, and identified them as having opposing morphologies. Additionally, the higher values of infection-related traits in LubWa1 indicate that its trichomes may have functioned as traps, limiting the spread of infection. Moreover, the CamBW1 genotype presented lower values for several chlorophyll fluorescence parameters during the early stages of drought, but at DP_4 (flowering), these trait values closely resembled those recorded under control conditions. Despite the stress impact, this genotype still maintained its yield potential. By including observations of multiple stress-induced responses of plants at six developmental stages in our experimental setup, we identified a critical moment—a trigger of dynamic changes in the plant’s stress response and the potential activation of such resistance mechanisms in CamBW1.

## Supplementary Information

Supplementary Material 1: Table S1. Description of the yield-forming traits with abbreviations.

Supplementary Material 2: Table S2. Traits associated with trichome micromorphology (with abbreviations) observed in this study.

Supplementary Material 3: Table S3. Design of gene-specific primers and evaluation of reference gene stability.

Supplementary Material 4: Table S4. ANOVA results – P values for testing significance of factor effects and their interaction (*F* test). 

Supplementary Material 5: Table S5. Significance of the interaction Treatment × DP for the studied genotypes (P < 0.05) and the sum of the significant interaction indicators.

Supplementary Material 6: Figure S1. Image acquisition and processing. The analysis was performed by using scanning electron micrographs at ×100 magnification. Image preprocessing steps: the images were converted from SEM microphotographs (A) to binary images (B); the processed image was subjected to auto calculation commend by Image Analyzing System Motic Images Plus 3.0 (C).

Supplementary Material 7: Figure S2. Mean values (with standard errors) of phenological traits for the studied genotypes grown under five treatments: control and combinations of drought (MD– mild; SD – severe) and foliar spray (GA – gibberellic acid, TR – trinexapac ethyl), DAS: days after sowing. Letters indicate statistically similar mean values at p < 0.05 according to the Fisher least significant difference test- lack of letters in cases of zero between-replicates variability 

Supplementary Material 8: Figure S3. The results were obtained from the Fusarium-induced effect evaluation method. (A) variability of the traits associated with fungal development (Fusarium biomass, % infected spikes, DON) observed for the four genotypes. Letters indicate statistically similar mean values at p < 0.05 according to the Fisher least significant difference test. (B) Scanning electron micrographs of the F. culmorum hypnea development observed on the inner side of the chaffs collected from plants subjected to four types of stress conditions. The colors in the diagrams represent experimental conditions: red – MD+F+GA; green – MD+F+TR; purple – SD+F+GA; yellow – SD+F+TR. 

Supplementary Material 9: Figure S4. Variability of trichome density observed for the four genotypes. Letters indicate statistically similar mean values at p < 0.05 according to the Fisher least significant difference test.

Supplementary Material 10: Figure S5. Differential curves of ∆Vt (double normalization) of four studied barley genotypes under different stress treatments at two development stages—DP_1 (A), DP_2 (B). The colors in the diagrams represent experimental conditions: black – control condition; red– MD+F+TR; green – MD+F+GA; blue – SD+F+TR; yellow – SD+F+GA. a.u. - arbitrary units.

Supplementary Material 11: Figure S6. Differential curves of ∆Vt (double normalization) of four studied barley genotypes under different stress treatments at two development stages—DP_3 (A), DP_4 (B). The colors in the diagrams represent experimental conditions: black – control condition; red– MD+F+TR; green – MD+F+GA; blue – SD+F+TR; yellow – SD+F+GA. a.u. - arbitrary units.

Supplementary Material 12: Figure S7. Differential curves of ∆Vt (double normalization) of four studied barley genotypes under different stress treatments at two development stages—DP_5 (A), DP_6 (B). The colors in the diagrams represent experimental conditions: black – control condition; red– MD+F+TR; green – MD+F+GA; blue – SD+F+TR; yellow – SD+F+GA. a.u. - arbitrary units. 

Supplementary Material 13: Figure S8. The variability of OJIP-derived parameters (measured in six development points - after exposure to drought and subsequent recovery) that characterize PSII functioning in four barley genotypes subjected to different treatments. Data (mean values with standard errors) are presented in arbitrary units. Treatments: black – control condition; red – MD+F+GA; green – MD+F+TR; purple – SD+F+GA; yellow – SD+F+TR.

Supplementary Material 14: Figure S9. Delayed fluorescence induction curves of the studied genotypes grown under optimal water conditions and subjected to stress combinations. Data (mean values with standard errors) are presented in arbitrary units. Treatments: black – control condition; red – MD+F+GA; green – MD+F+TR; purple – SD+F+GA; yellow – SD+F+TR. 

Supplementary Material 15: Figure S10. Kinetics of modulated light reflection at 820 nm of the studied genotypes grown under optimal water conditions and subjected to stress combinations. Data (mean values with standard errors) are presented in arbitrary units. Treatments: black – control condition; red – MD+F+GA; green – MD+F+TR; purple – SD+F+GA; yellow – SD+F+TR. 

Supplementary Material 16: Figure S11. Flavonol index (Flav) and anthocyanin index (Anth) (measured in six development points - after exposure to drought and subsequent recovery) of the studied genotypes grown under optimal water conditions and subjected to stress combinations. Data (mean values with standard errors) are presented in arbitrary units.

Supplementary Material 17: Figure S12. Distribution of traits associated with tillering process and grain yield. Letters indicate statistically similar mean values at p < 0.05 according to the Fisher least significant difference test. 

Supplementary Material 18: Figure S13. Distribution of traits associated with main spike morphology. Letters indicate statistically similar mean values at p < 0.05 according to the Fisher least significant difference test.

Supplementary Material 19: Figure S14. Distribution of traits associated with lateral spike morphology. Letters indicate statistically similar mean values at p < 0.05 according to the Fisher least significant difference test. 

## Data Availability

All data generated or analyzed during this study are included in this published article [and its supplementary information files].

## Abbreviations

LFE: Last flag extension
DP_1: Development point 1
DP_2: Development point 2
DP_3: Development point 3
DP_4: Development point 4
DP_5: Development point 5
DP_6: Development point 6
MFSC: Multifactorial stress combination
GA3: Gibberellic acid
TR: Trinexapac-ethyl
DON: Deoxynivalenol
Chl: Chlorophyll
Flav: Flavonoids
Anth: Anthocyanins
NBI: Nitrogen balance index
HvPRR37: Hordeum vulgare Pseudo-Response Regulator 37
PSI: Photosystem I
PSII: Photosystem II
FHB: Fusarium head blight
