# Supplementary material for: Effect of developmental dynamics on WRKY expression in barley with varying phenologies and trichome micromorphologies
Source: BMC Plant Biol. 2025 Dec 17;26:109. doi: 10.1186/s12870-025-07933-5 (PMC12822057; doi:10.1186/s12870-025-07933-5)
Supplement: Supplementary file 1 — Supplementary Material 1: Table S1. Description of the yield-forming traits with abbreviations. [file 12870_2025_7933_MOESM1_ESM.docx]

**Table S1.** Description of the yield-forming traits with abbreviations

| **Trait (Unit), (Abbrev.)** | **Trait Description** |
| --- | --- |
| Total number of tillers (Tn) | Number of tillers with fertile and non-fertile (without grains) spikes (observations were carried out for all pots within each treatment) |
| Number of productive tillers (PTn) | Number of tillers with fertile spikes (observations were carried out for all pots within each treatment) |
| Length of main spike (cm), (LSm) | Length of main spike from 10 randomly selected spikes in a pot (without awns) |
| Number of spikelets per main spike, (NSSm) | Number of spikelets in spike of main stem-average e for 10 main spikes in a pot |
| Number of grains per main spike, (NGSm) | Number of grains collected from one spike of main stem—average for 10 main spikes in a pot |
| Weight of grains per main spike (g), (WGSm) | Weight of grain collected from one spike of the main stem—average for 10 main spikes in a pot |
| Length of lateral spike (cm), (LSl) | Length of spike from lateral stem—average for 10 lateral spikes in a pot (without awns) |
| Number of spikelets per lateral spike, (NSSl) | Number of spikelets per spike of lateral stem—average for 10 lateral spikes in a pot |
| Number of grains per lateral spike, (NGSl) | Number of grains collected from spike of lateral stem—average for 10 lateral spikes in a pot |
| Weight of grains per main spike (g), (WGSl) | Weight of grain collected from one spike of the lateral stem—average for 10 lateral spikes in a pot |
| Grain yield (g), (GY) | Average weight of grains collected from one plant, calculated as average of measurements of grain weight for 10 plants |
| Thousand grain weight (g), (TGW) | Average weight of 1000 grains, calculated as average of 1000 × average weight of one grain for 20 spikes in a pot |
