# Supplementary material for: Effect of developmental dynamics on WRKY expression in barley with varying phenologies and trichome micromorphologies
Source: BMC Plant Biol. 2025 Dec 17;26:109. doi: 10.1186/s12870-025-07933-5 (PMC12822057; doi:10.1186/s12870-025-07933-5)
Supplement: Supplementary file 2 — Supplementary Material 2: Table S2. Traits associated with trichome micromorphology (with abbreviations) observed in this study. [file 12870_2025_7933_MOESM2_ESM.docx]

**Table S2**. Traits associated with trichome micromorphology (with abbreviations) observed in this study

| **Trait** | **Abbreviation** |
| --- | --- |
| Object number | Ono |
| Percent of object area in picture area | % obj area |
| Max object area (µm^2^) | Max area |
| Mean object area (µm^2^) | Mean area |
| Max object perimenter (µm) | Max perimenter |
| Mean object perimenter (µm) | Mean perimenter |
