# Supplementary material for: Effect of developmental dynamics on WRKY expression in barley with varying phenologies and trichome micromorphologies
Source: BMC Plant Biol. 2025 Dec 17;26:109. doi: 10.1186/s12870-025-07933-5 (PMC12822057; doi:10.1186/s12870-025-07933-5)
Supplement: Supplementary file 3 — Supplementary Material 3: Table S3. Design of gene-specific primers and evaluation of reference gene stability. [file 12870_2025_7933_MOESM3_ESM.docx]

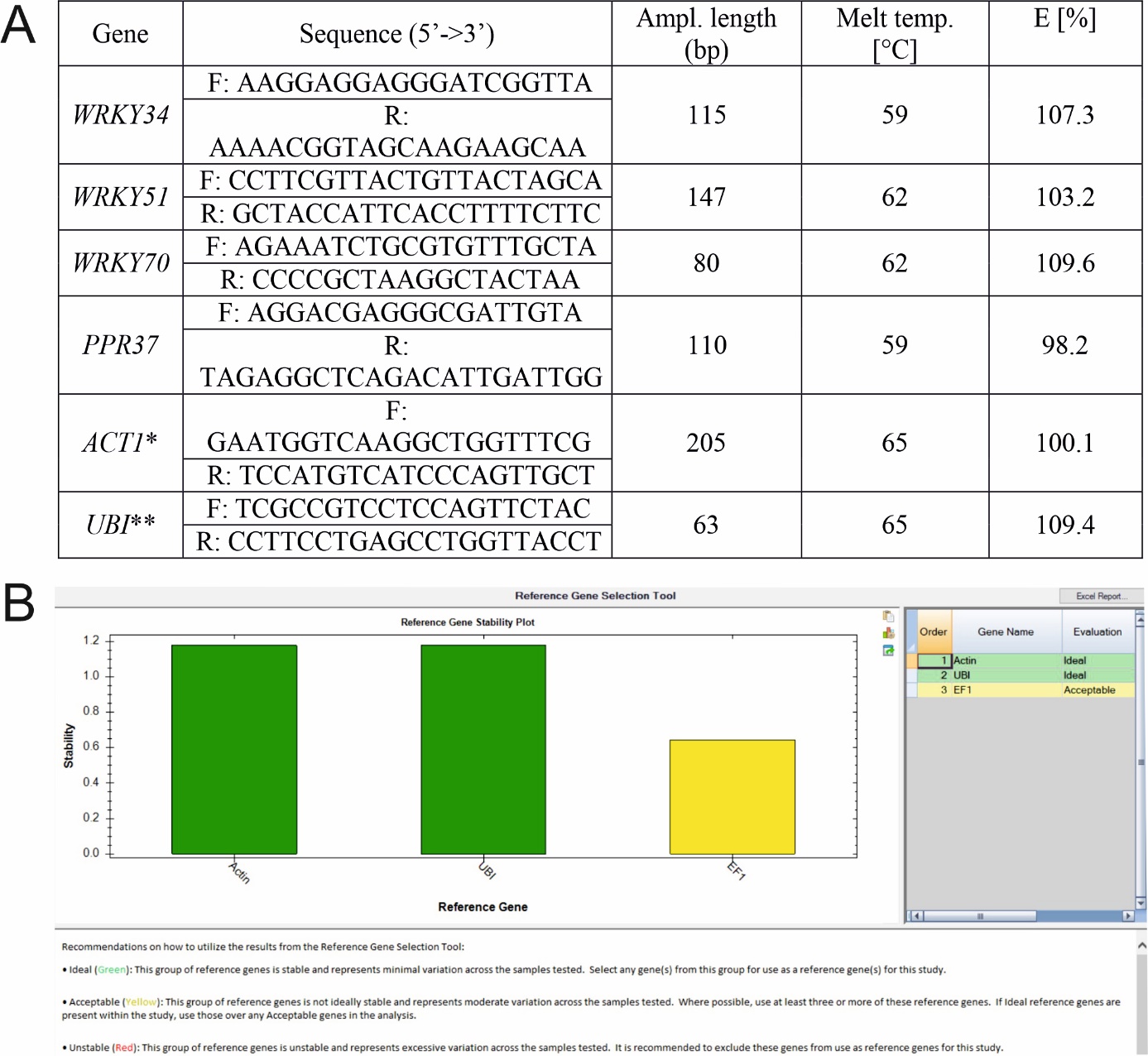


**Table S3**. Design of gene-specific primers and evaluation of reference gene stability. (A) DNA sequences of the gene specific primers (* Gines et al., 2018; DOI: 10.2135/cropsci2017.07.0443, ** Rapacz et al., 2012; DOI: 10.1007/s11738-012-0967-1). (B) In this study, three candidate reference genes were evaluated for normalization. Based on the stability analysis performed using the Reference Gene Selection Tool (Bio-Rad CFX Maestro Software), two reference genes showing the most stable expression were selected
