# Supplementary material for: Effect of developmental dynamics on WRKY expression in barley with varying phenologies and trichome micromorphologies
Source: BMC Plant Biol. 2025 Dec 17;26:109. doi: 10.1186/s12870-025-07933-5 (PMC12822057; doi:10.1186/s12870-025-07933-5)
Supplement: Supplementary file 4 — Supplementary Material 4: Table S4. ANOVA results – P values for testing significance of factor effects and their interaction (F test). [file 12870_2025_7933_MOESM4_ESM.docx]

**Table S4**. ANOVA results – P values for testing significance of factor effects and their interaction (*F* test)

| **Type of the traits** | **Name of the trait** | **Genotype (G)** | **Treatment (T)** | **G × T interaction** |
| --- | --- | --- | --- | --- |
| Phenotype | Tn | 0.253 | < 0.001 | 0.095 |
|  | PTn | 0.340 | 0.254 | 0.924 |
|  | LSl | 0.284 | < 0.001 | 0.025 |
|  | NSSl | 0.272 | < 0.001 | 0.010 |
|  | NGSl | 0.019 | < 0.001 | 0.313 |
|  | WGSl | 0.257 | < 0.001 | 0.250 |
|  | LSm | 0.353 | < 0.001 | 0.195 |
|  | NSSm | 0.349 | < 0.001 | 0.056 |
|  | NGSm | 0.865 | < 0.001 | 0.111 |
|  | WGSm | 0.704 | < 0.001 | 0.067 |
|  | GY | 0.701 | < 0.001 | 0.311 |
|  | TGW | 0.132 | 0.008 | 0.003 |
|  |  |  |  |  |
| Phenology | tillering^a)^ | - | - | - |
|  | flag_leaf_stage | < 0.001 | < 0.001 | < 0.001 |
|  | flowering | < 0.001 | < 0.001 | < 0.001 |
|  | heading | < 0.001 | < 0.001 | < 0.001 |
|  |  |  |  |  |
| Trichome morphology | Ono | 0.001 |  |  |
|  | %_obj_area | 0.113 |  |  |
|  | Max_area | 0.244 |  |  |
|  | Mean_area | 0.013 |  |  |
|  | Max_perimeter | 0.174 |  |  |
|  | Mean_perimeter | 0.065 |  |  |
|  |  |  |  |  |
| Fusarium infection evaluation | Fusarium_biomass | 0.572 | 0.110 | 0.117 |
|  | %_infected_spikes | 0.102 | 0.353 | 0.760 |
|  | DON | 0.083 | 0.201 | 0.199 |
|  | density | 0.489 | < 0.001 | 0.001 |

^a)^ Testing was not performed due to lack of variation
