# Supplementary material for: Effect of developmental dynamics on WRKY expression in barley with varying phenologies and trichome micromorphologies
Source: BMC Plant Biol. 2025 Dec 17;26:109. doi: 10.1186/s12870-025-07933-5 (PMC12822057; doi:10.1186/s12870-025-07933-5)
Supplement: Supplementary file 5 — Supplementary Material 5: Table S5. Significance of the interaction Treatment × DP for the studied genotypes (P < 0.05) and the sum of the significant interaction indicators. [file 12870_2025_7933_MOESM5_ESM.docx]

**Table S5**. Significance of the interaction Treatment × DP for the studied genotypes (P < 0.05) and the sum of the significant interaction indicators

| **Trait** | **Number of significant treatment × DP interaction** | | | | **P-value** | | | |
| --- | --- | --- | --- | --- | --- | --- | --- | --- |
|  | CamBW1 | CamWa1 | LubBW1 | LubWa1 | CamBW1 | CamWa1 | LubBW1 | LubWa1 |
| RE_0_/RC | 1 | 0 | 0 | 0 | 0.05 | 0.831 | 0.096 | 0.303 |
| ϕ_Po_ | 1 | 0 | 0 | 0 | 0.026 | 0.626 | 0.135 | 0.15 |
| δ_Ro_ | 1 | 0 | 0 | 0 | 0.013 | 0.884 | 0.189 | 0.444 |
| ϕ_Ro_ | 1 | 0 | 0 | 0 | 0.007 | 0.607 | 0.447 | 0.558 |
| ϕ_Do_ | 1 | 0 | 0 | 0 | 0.026 | 0.626 | 0.135 | 0.15 |
| PI_total_ | 1 | 0 | 0 | 0 | 0.002 | 0.269 | 0.576 | 0.727 |
| I_2_ | 0 | 1 | 0 | 0 | 0.061 | 0.023 | 0.579 | 0.389 |
| Mr_max_ | 1 | 0 | 0 | 0 | 0.008 | 0.591 | 0.56 | 0.538 |
| Chl | 0 | 1 | 1 | 0 | 0.599 | 0.026 | 0.012 | 0.069 |
| NBI | 0 | 0 | 1 | 1 | 0.253 | 0.202 | 0.046 | 0.022 |
| WRKY34 | 1 | 1 | 1 | 1 | 0.026 | 0.006 | <0.001 | 0.017 |
| WRKY51 | 1 | 0 | 0 | 1 | 0.012 | 0.633 | 0.054 | 0.002 |
| WRKY70 | 1 | 1 | 1 | 1 | <0.001 | 0.044 | 0.002 | 0.02 |
| **total** | 10 | 4 | 4 | 4 |  |  |  |  |
