# Supplementary material for: Effect of developmental dynamics on WRKY expression in barley with varying phenologies and trichome micromorphologies
Source: BMC Plant Biol. 2025 Dec 17;26:109. doi: 10.1186/s12870-025-07933-5 (PMC12822057; doi:10.1186/s12870-025-07933-5)
Supplement: Supplementary file 6 — Supplementary Material 6: Figure S1. Image acquisition and processing. The analysis was performed by using scanning electron micrographs at ×100 magnification. Image preprocessing steps: the images were converted from SEM microphotographs (A) to binary images (B); the processed image was subjected to auto calculation commend by Image Analyzing System Motic Images Plus 3.0 (C). [file 12870_2025_7933_MOESM6_ESM.docx]

**
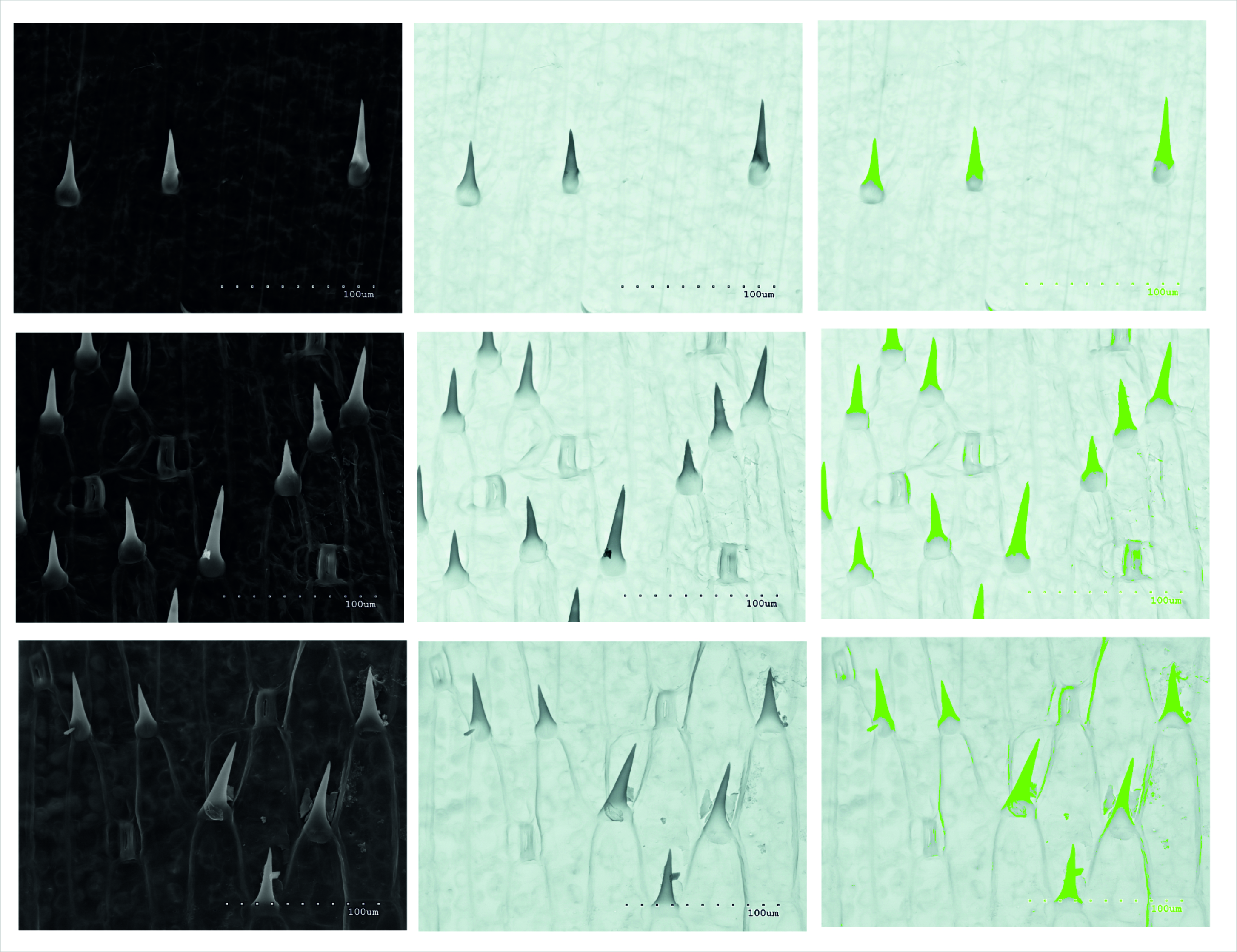
Figure S1**. Image acquisition and processing. The analysis was performed by using scanning electron micrographs at ×100 magnification. Image preprocessing steps: the images were converted from SEM microphotographs (A) to binary images (B); the processed image was subjected to auto calculation commend by Image Analyzing System Motic Images Plus 3.0 (C)
