# Supplementary material for: Effect of developmental dynamics on WRKY expression in barley with varying phenologies and trichome micromorphologies
Source: BMC Plant Biol. 2025 Dec 17;26:109. doi: 10.1186/s12870-025-07933-5 (PMC12822057; doi:10.1186/s12870-025-07933-5)
Supplement: Supplementary file 7 — Supplementary Material 7: Figure S2. Mean values (with standard errors) of phenological traits for the studied genotypes grown under five treatments: control and combinations of drought (MD– mild; SD – severe) and foliar spray (GA – gibberellic acid, TR – trinexapac ethyl), DAS: days after sowing. Letters indicate statistically similar mean values at p < 0.05 according to the Fisher least significant difference test- lack of letters in cases of zero between-replicates variability [file 12870_2025_7933_MOESM7_ESM.docx]

**Figure S2**. Mean values (with standard errors) of phenological traits for the studied genotypes grown under five treatments: control and combinations of drought (MD – mild; SD – severe) and foliar spray (GA – gibberellic acid, TR – trinexapac ethyl), DAS: days after sowing. Letters indicate statistically similar mean values at p < 0.05 according to the Fisher least significant difference test - lack of letters in cases of zero between-replicates variability
