# Supplementary material for: Effect of developmental dynamics on WRKY expression in barley with varying phenologies and trichome micromorphologies
Source: BMC Plant Biol. 2025 Dec 17;26:109. doi: 10.1186/s12870-025-07933-5 (PMC12822057; doi:10.1186/s12870-025-07933-5)
Supplement: Supplementary file 10 — Supplementary Material 10: Figure S5. Differential curves of ∆Vt (double normalization) of four studied barley genotypes under different stress treatments at two development stages—DP_1 (A), DP_2 (B). The colors in the diagrams represent experimental conditions: black – control condition; red– MD+F+TR; green – MD+F+GA; blue – SD+F+TR; yellow – SD+F+GA. a.u. - arbitrary units. [file 12870_2025_7933_MOESM10_ESM.docx]

**Figure S5**. Differential curves of ∆Vt (double normalization) of four studied barley genotypes under different stress treatments at two development stages—DP_1 (A), DP_2 (B). The colors in the diagrams represent experimental conditions: **black** – control condition; **red** – MD+F+TR; **green** – MD+F+GA; **blue** – SD+F+TR; **yellow** – SD+F+GA. a.u. - arbitrary units
