# Supplementary material for: Effect of developmental dynamics on WRKY expression in barley with varying phenologies and trichome micromorphologies
Source: BMC Plant Biol. 2025 Dec 17;26:109. doi: 10.1186/s12870-025-07933-5 (PMC12822057; doi:10.1186/s12870-025-07933-5)
Supplement: Supplementary file 13 — Supplementary Material 13: Figure S8. The variability of OJIP-derived parameters (measured in six development points - after exposure to drought and subsequent recovery) that characterize PSII functioning in four barley genotypes subjected to different treatments. Data (mean values with standard errors) are presented in arbitrary units. Treatments: black – control condition; red – MD+F+GA; green – MD+F+TR; purple – SD+F+GA; yellow – SD+F+TR. [file 12870_2025_7933_MOESM13_ESM.docx]

**
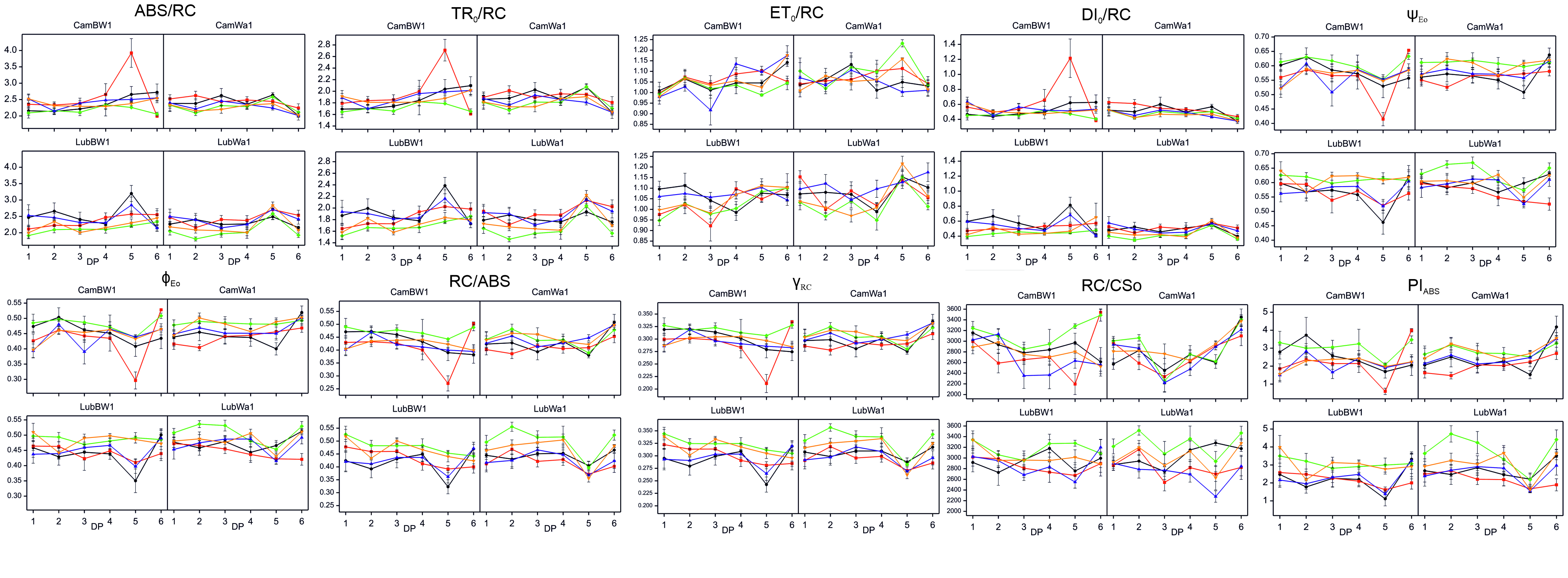
Figure S8**. The variability of OJIP-derived parameters (measured in six development points - after exposure to drought and subsequent recovery) that characterize PSII functioning in four barley genotypes subjected to different treatments. Data (mean values with standard errors) are presented in arbitrary units. Treatments: **black** – control condition; **red** – MD+F+GA; **green** – MD+F+TR; **purple** – SD+F+GA; **yellow** – SD+F+TR
