# Supplementary material for: Effect of developmental dynamics on WRKY expression in barley with varying phenologies and trichome micromorphologies
Source: BMC Plant Biol. 2025 Dec 17;26:109. doi: 10.1186/s12870-025-07933-5 (PMC12822057; doi:10.1186/s12870-025-07933-5)
Supplement: Supplementary file 15 — Supplementary Material 15: Figure S10. Kinetics of modulated light reflection at 820 nm of the studied genotypes grown under optimal water conditions and subjected to stress combinations. Data (mean values with standard errors) are presented in arbitrary units. Treatments: black – control condition; red – MD+F+GA; green – MD+F+TR; purple – SD+F+GA; yellow – SD+F+TR. [file 12870_2025_7933_MOESM15_ESM.docx]

**Figure S10**. Kinetics of modulated light reflection at 820 nm of the studied genotypes grown under optimal water conditions and subjected to stress combinations. Data (mean values with standard errors) are presented in arbitrary units. Treatments: **black** – control condition; **red** – MD+F+GA; **green** – MD+F+TR; **purple** – SD+F+GA; **yellow** – SD+F+TR
