# Supplementary material for: Effect of developmental dynamics on WRKY expression in barley with varying phenologies and trichome micromorphologies
Source: BMC Plant Biol. 2025 Dec 17;26:109. doi: 10.1186/s12870-025-07933-5 (PMC12822057; doi:10.1186/s12870-025-07933-5)
Supplement: Supplementary file 16 — Supplementary Material 16: Figure S11. Flavonol index (Flav) and anthocyanin index (Anth) (measured in six development points - after exposure to drought and subsequent recovery) of the studied genotypes grown under optimal water conditions and subjected to stress combinations. Data (mean values with standard errors) are presented in arbitrary units. [file 12870_2025_7933_MOESM16_ESM.docx]

**Figure S11**. Flavonol index (Flav) and anthocyanin index (Anth) (measured in six development points - after exposure to drought and subsequent recovery) of the studied genotypes grown under optimal water conditions and subjected to stress combinations. Data (mean values with standard errors) are presented in arbitrary units
