# Supplementary material for: Effect of developmental dynamics on WRKY expression in barley with varying phenologies and trichome micromorphologies
Source: BMC Plant Biol. 2025 Dec 17;26:109. doi: 10.1186/s12870-025-07933-5 (PMC12822057; doi:10.1186/s12870-025-07933-5)
Supplement: Supplementary file 17 — Supplementary Material 17: Figure S12. Distribution of traits associated with tillering process and grain yield. Letters indicate statistically similar mean values at p < 0.05 according to the Fisher least significant difference test. [file 12870_2025_7933_MOESM17_ESM.docx]

**Figure S12**. Distribution of traits associated with tillering process and grain yield. Letters indicate statistically similar mean values at p < 0.05 according to the Fisher least significant difference test
