# Supplementary material for: Effect of developmental dynamics on WRKY expression in barley with varying phenologies and trichome micromorphologies
Source: BMC Plant Biol. 2025 Dec 17;26:109. doi: 10.1186/s12870-025-07933-5 (PMC12822057; doi:10.1186/s12870-025-07933-5)
Supplement: Supplementary file 19 — Supplementary Material 19: Figure S14. Distribution of traits associated with lateral spike morphology. Letters indicate statistically similar mean values at p < 0.05 according to the Fisher least significant difference test. [file 12870_2025_7933_MOESM19_ESM.docx]

**Figure S14**. Distribution of traits associated with lateral spike morphology. Letters indicate statistically similar mean values at p < 0.05 according to the Fisher least significant difference test
