# Supplementary material for: Effect of developmental dynamics on WRKY expression in barley with varying phenologies and trichome micromorphologies
Source: BMC Plant Biol. 2025 Dec 17;26:109. doi: 10.1186/s12870-025-07933-5 (PMC12822057; doi:10.1186/s12870-025-07933-5)
Supplement: Supplementary file 8 — Supplementary Material 8: Figure S3. The results were obtained from the Fusarium-induced effect evaluation method. (A) variability of the traits associated with fungal development (Fusarium biomass, % infected spikes, DON) observed for the four genotypes. Letters indicate statistically similar mean values at p < 0.05 according to the Fisher least significant difference test. (B) Scanning electron micrographs of the F. culmorum hypnea development observed on the inner side of the chaffs collected from plants subjected to four types of stress conditions. The colors in the diagrams represent experimental conditions: red – MD+F+GA; green – MD+F+TR; purple – SD+F+GA; yellow – SD+F+TR. [file 12870_2025_7933_MOESM8_ESM.docx]

**Figure S3**. The results were obtained from the *Fusarium*-induced effect evaluation method. (A) variability of the traits associated with fungal development (Fusarium biomass, % infected spikes, DON) observed for the four genotypes. Letters indicate statistically similar mean values at p < 0.05 according to the Fisher least significant difference test. (B) Scanning electron micrographs of the *F. culmorum* hypnea development observed on the inner side of the chaffs collected from plants subjected to four types of stress conditions. The colors in the diagrams represent experimental conditions: **red** – MD+F+GA; **green** – MD+F+TR; **purple** – SD+F+GA; **yellow** – SD+F+TR
